# Integrated Multi‐Omics and Radiogenomic Analysis Identifies S100B and ITGB5 as Complementary Candidate Biomarkers of Glioblastoma Heterogeneity

**DOI:** 10.1111/cbdd.70396

**Published:** 2026-09-14

**Authors:** Xuhang Yang, Yusong Zhang, Yutuo Zheng, Jian Lin

**Affiliations:** ^1^ Wenzhou Medical University Wenzhou China; ^2^ Department of Neurosurgery The Second Affiliated Hospital of Wenzhou Medical University Wenzhou China; ^3^ The Key Laboratory of Pediatric Hematology and Oncology Diseases of Wenzhou The Second Affiliated Hospital of Wenzhou Medical University Wenzhou China

**Keywords:** glioblastoma, heterogeneity, ITGB5, microbiota‐associated genes, multi‐omics, radiogenomics, S100B, single‐cell transcriptomics, spatial transcriptomics, tumor microenvironment

## Abstract

Glioblastoma (GBM) is characterized by marked intratumoral heterogeneity, diffuse invasion, and a profoundly immunosuppressive tumor microenvironment. In this study, we applied an integrative multi‐omics and radiogenomic framework to identify candidate biomarkers reflecting complementary dimensions of GBM heterogeneity. Public bulk transcriptomic datasets were integrated to define GBM‐related differentially expressed genes, which were intersected with curated microbiota‐associated gene sets as a hypothesis‐generating screening strategy. Machine‐learning models with SHAP interpretation were used for candidate‐gene prioritization, followed by survival‐association analysis, pan‐cancer comparison, Human Protein Atlas immunohistochemistry, CPTAC proteomics, immune infiltration analysis, single‐cell transcriptomics, spatial transcriptomics, in silico perturbation analysis, and MRI‐based radiogenomics. GBM samples showed enrichment of extracellular matrix organization, proliferative programs, immune‐related signaling, and vascular or endothelial pathways, with relative reductions in neural, synaptic, and myelin‐associated signatures. S100B and ITGB5 emerged as survival‐associated candidate markers with different biological contexts. Multi‐omics analyses suggested that S100B may reflect broadly distributed glial‐lineage and malignant‐state programs, whereas ITGB5 was more closely associated with focal extracellular matrix remodeling, stromal‐vascular interactions, and immunoregulatory niches. Radiogenomic analysis further suggested distinct MRI‐derived associations for the two genes, with the ITGB5‐related model retaining a broader radiomic signature and showing a more heterogeneous habitat pattern than the S100B‐related model. These exploratory findings support S100B and ITGB5 as complementary candidate biomarkers of GBM heterogeneity and provide a basis for future experimental, multicenter, and prospective validation.

## Graphical abstract

An integrative multi‐omics and radiogenomic framework identifies S100B and ITGB5 as complementary biomarkers of glioblastoma heterogeneity. S100B reflects broad glial–malignant programs, whereas ITGB5 marks focal stromal–vascular, immunoregulatory, and radiomic heterogeneity.

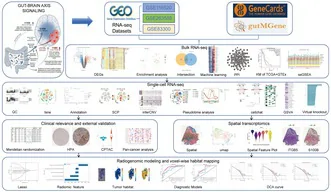

## 1 Introduction

Glioblastoma (GBM) is the most aggressive primary tumor of the central nervous system (Tang et al. 2025). Even with maximal safe resection followed by radiotherapy and temozolomide‐based chemotherapy, the prognosis remains poor and most patients eventually experience recurrence (Brown et al. 2020; McAleavey et al. 2022). This unfavorable clinical course is closely related to the marked biological heterogeneity of GBM. Such heterogeneity is not confined to malignant tumor cells but also involves a complex tumor microenvironment (TME), where immune, vascular, stromal, and glial‐lineage components interact with tumor cells in a dynamic and spatially organized manner (Jang and Park 2025; Crivii et al. 2022).

The GBM microenvironment contains glioma stem‐like cells, glioma‐associated macrophages and microglia, myeloid‐derived suppressor cells, endothelial cells, stromal components, and other immune cell populations (Jain et al. 2022; Jackson et al. 2023). These cellular components communicate through cytokines, chemokines, extracellular matrix molecules, vascular signals, and metabolic mediators, thereby contributing to immune escape, diffuse invasion, angiogenesis, and therapeutic resistance (Tang et al. 2025; Liu et al. 2025; Aydin et al. 2024; Gagliardi et al. 2025). This complex and immunosuppressive ecosystem may partly explain why immune checkpoint inhibitors have shown limited efficacy in GBM, despite their success in several other solid tumors (Jain et al. 2022; Surendran et al. 2023). Therefore, understanding GBM requires an integrated view of tumor‐cell states, microenvironmental remodeling, spatial organization, and systemic regulatory contexts.

In addition to local microenvironmental regulation, systemic factors may participate in shaping GBM‐associated inflammatory and metabolic states. The gut–brain axis has received increasing attention as a potential link between intestinal microbiota, central nervous system inflammation, immune regulation, and metabolic homeostasis (Cryan et al. 2019). Microbial communities and their metabolites may affect blood–brain barrier integrity, microglial activation, and neuroinflammatory responses through neural, endocrine, metabolic, and immune pathways (Zhao et al. 2025; Böckels et al. 2026). Emerging studies have suggested that gut dysbiosis may influence glioma progression and treatment response by reshaping systemic immune status (Krawczyk et al. 2025; Scuderi et al. 2025; Zhang et al. 2024). However, current evidence remains largely indirect, and the present study did not include microbiome, metagenomic, metabolomic, or microbiota‐perturbation measurements. Therefore, microbiota‐associated genes were used here only as a hypothesis‐generating candidate pool rather than as evidence of direct microbiota‐driven regulation in GBM.

Recent single‐cell and spatial transcriptomic studies have shown that GBM should not be viewed as a uniform tumor mass. Instead, it is composed of distinct malignant‐cell states and spatially organized microenvironmental niches (Ravi et al. 2022; Greenwald et al. 2024; Lv et al. 2024; Ruiz‐Moreno et al. 2025). These approaches provide important information on tumor‐cell plasticity, immune–stromal interactions, and local tissue organization. Nevertheless, they depend on tumor tissue sampling and are difficult to apply repeatedly during clinical follow‐up. By contrast, magnetic resonance imaging (MRI) is routinely used in GBM management. Radiomics and radiogenomic analyses have suggested that some molecular and microenvironmental features of GBM may be reflected by imaging‐derived phenotypes (Wang et al. 2025; Do et al. 2022; Kandalgaonkar et al. 2022; Tang et al. 2024; Marasi et al. 2026; Fathi Kazerooni et al. 2025). Thus, biomarkers that connect molecular alterations with cellular distribution, spatial localization, and imaging features may help clarify complementary dimensions of GBM heterogeneity beyond conventional bulk transcriptomic analysis.

S100B and ITGB5 are two candidate molecules with different biological implications in GBM. S100B is a calcium‐binding protein mainly expressed in glial‐lineage cells and is commonly associated with neural injury, astroglial activation, and neuroinflammation (Dali et al. 2025). Previous studies have also linked S100B to glioma‐associated microglial/macrophage regulation and inflammatory signaling within the tumor context (Zhang et al. 2011; Wang et al. 2013). ITGB5, in contrast, belongs to the integrin family and participates in cell–extracellular matrix interaction. ITGB5 has been implicated in tumor progression across several cancer types, including GBM (Chen et al. 2025; Cheng et al. 2024; Zhang et al. 2019). These findings suggest a biologically meaningful contrast: S100B may reflect a relatively broad glial lineage, malignant state, and spatial ecological context, whereas ITGB5 may be more closely associated with extracellular matrix remodeling, stromal‐vascular interaction, and immune‐microenvironmental architecture.

Current GBM biomarker studies still have several limitations. Bulk transcriptomic analyses provide averaged expression profiles and cannot determine which cell populations or spatial regions mainly contribute to a given signal. Expression‐ or prognosis‐based screening alone also has limited ability to distinguish biomarkers reflecting widespread tumor‐state programs from those associated with focal microenvironmental niches. Although S100B and ITGB5 have been reported in glioma‐related expression, prognostic, immune, or functional studies (Zhang et al. 2011, 2019; Wang et al. 2013), they have not been systematically evaluated across transcriptomic, protein‐level, immune, cellular, spatial, perturbation‐related, and radiographic layers. A combined framework incorporating bulk transcriptomics, machine learning, protein‐level validation, immune profiling, single‐cell transcriptomics, spatial transcriptomics, in silico perturbation analysis, and radiogenomic modeling may provide a practical way to examine whether these genes represent distinct dimensions of GBM heterogeneity.

In this study, we applied an integrative multi‐omics and radiogenomic framework to determine whether S100B and ITGB5 represent complementary dimensions of GBM heterogeneity. We examined these two candidate biomarkers across transcriptomic, protein‐level, immune, single‐cell, spatial, perturbation‐related, and MRI‐derived imaging layers. Through this framework, we evaluated their diagnostic‐model contribution, survival association, cellular and spatial distribution, functional context, immune‐microenvironmental associations, and noninvasive imaging correlates. Our findings provide a cautious multidimensional interpretation of S100B and ITGB5 in GBM and may support future functional, molecular, and imaging‐based validation studies.

## 2 Methods

### 2.1 Data Acquisition and Preprocessing

Publicly available datasets were collected to investigate transcriptomic changes, tumor heterogeneity, and imaging‐related molecular features in GBM. Bulk transcriptomic datasets were obtained from the Gene Expression Omnibus (GEO) database, with GSE116520, GSE263588, and GSE83300 used as discovery cohorts, and GSE119834 and GSE137900 used for external validation. When available, transcriptomic, clinical, and imaging information from The Cancer Genome Atlas (TCGA) and the Chinese Glioma Genome Atlas (CGGA) were also included for validation and downstream analyses. Single‐cell RNA sequencing data from GSE135045 were used to examine cellular composition and malignant‐cell transcriptional states, while spatial transcriptomic data from three GBM samples in GSE237183, including GSM7596588, GSM7596591, and GSM7596593, were used to evaluate the spatial distribution of selected genes. For bulk transcriptomic analysis, genes shared by the included datasets were matched according to official gene symbols, and the corresponding expression matrices were subsequently merged. Batch effects caused by dataset source were adjusted using the removeBatchEffect function in the limma package. Principal component analysis (PCA) was used to assess the effect of batch correction, and sample‐level expression distributions before and after correction were also compared. Unless otherwise stated, all statistical analyses were performed in R.

### 2.2 Identification of Microbiota‐Associated Candidate Genes and Bulk Transcriptomic Analysis

Differential expression analysis was performed using the limma package. Genes with an absolute log_2_ fold change > 0.5 and an adjusted *p* value < 0.05 were considered differentially expressed genes (DEGs) and were used for subsequent functional enrichment and candidate‐gene screening. The microbiota‐associated gene set was constructed as a hypothesis‐generating candidate pool and was not intended to demonstrate direct microbial regulation of GBM gene expression. Microbiota‐relevant genes were collected from GeneCards and gutMGene. GeneCards was searched on January 3, 2026, using the predefined search term “Microbial metabolite”. Genes with a GeneCards relevance score > 2.0 were retained, and this relevance‐score threshold was used as the only filtering criterion. No additional restriction to protein‐coding genes was applied. Gut microbiota‐related host genes were obtained from gutMGene on January 3, 2026. Curated host gene–microbe and host gene–microbial metabolite association entries were retrieved, and only *Homo sapiens* host‐gene records were retained. Host genes were extracted from the curated association entries and mapped to official gene symbols. After duplicate removal, the GeneCards‐derived and gutMGene‐derived genes were combined to generate the final microbiota‐associated candidate gene set.

The overlap between this microbiota‐associated candidate gene set and GBM‐related DEGs was used for downstream prioritization. This overlap‐based strategy was interpreted as identifying microbiota‐associated or microbiota‐relevant candidate genes within GBM‐related transcriptional alterations, rather than as evidence that these genes were directly regulated by microbiota in GBM. Functional relationships among the overlapping candidate genes were examined using GeneMANIA and were interpreted as database‐derived functional associations rather than as experimentally verified regulatory interactions. To characterize the biological processes represented by GBM‐related transcriptional alterations, Gene Ontology (GO), Kyoto Encyclopedia of Genes and Genomes (KEGG), Disease Ontology (DO), and Reactome enrichment analyses were performed using the clusterProfiler. Gene set enrichment analysis (GSEA) was also conducted to identify pathways showing coordinated transcriptomic changes, and significant enrichment terms were further grouped and visualized using aPEAR to summarize major functional themes. Single‐sample gene set enrichment analysis (ssGSEA) was used to estimate immune‐cell infiltration and immune‐related functional signatures in each sample. Differences in immune signatures between control and GBM tissues were compared, and correlations between target‐gene expression and immune‐related scores were evaluated using Spearman correlation analysis.

### 2.3 Machine Learning‐Based Diagnostic Modeling and Interpretability Analysis

Machine‐learning models were constructed to evaluate the diagnostic‐model contribution of the candidate genes. The 83 genes identified by intersecting the GBM‐related differentially expressed genes with the microbiota‐associated candidate‐gene set were used as input features. The merged discovery cohort was used for feature selection and model training, whereas GSE119834 and GSE137900 were retained as independent external validation cohorts. These external cohorts were not involved in feature selection, hyperparameter tuning, or model optimization. Expression matrices from the discovery and validation cohorts were processed separately, and centering and scaling were performed within each cohort to account for cohort‐specific differences in expression scale.

A two‐stage modeling strategy was adopted. First, algorithms with embedded or model‐based feature‐selection capacity were used to identify informative genes. Second, the retained genes were incorporated into multiple classifiers for diagnostic‐model construction. Fifteen baseline algorithms covering regularized regression, stepwise generalized linear models, boosting methods, tree‐based models, kernel‐based classifiers, discriminant analysis, instance‐based learning, and Bayesian classifiers were evaluated. In total, 175 feature‐selection and classification combinations were tested. The random seed was set to 123. Models retaining three or fewer genes were excluded, and the maximum number of retained genes was restricted to 15. For algorithms requiring internal tuning, cross‐validation was performed within the discovery cohort according to the corresponding model‐specific settings. Model performance was evaluated using the area under the receiver operating characteristic curve (AUC), and model consistency was assessed across the discovery and independent validation cohorts. The model showing the best overall performance with consistent validation results was selected for subsequent interpretation. Detailed parameter settings, validation strategies, and output metrics are provided in Table S1.

For model interpretability, SHAP‐based analysis was performed using the prioritized 15‐gene expression matrix. Samples were stratified into a training set and an internal validation set at a ratio of 7:3 using createDataPartition and a random seed of 12,345. Five classifiers, including random forest, support vector machine, XGBoost, gradient boosting machine, and k‐nearest neighbors, were trained using 5‐fold resampling within the caret framework. Receiver operating characteristic curves, AUC values, and corresponding 95% confidence intervals were calculated in the internal validation set using the pROC package. The classifier with the highest internal validation AUC was selected for subsequent SHAP‐based interpretation. Because the internal validation set was used for model comparison and selection, it was not regarded as an independent external test set.

Kernel SHAP values for the selected fitted model were calculated using the training‐set expression matrix and the kernelshap package, and the results were visualized using shapviz. Features were ranked according to their mean absolute SHAP values, and all 15 retained genes were reported. SHAP importance bar plots, beeswarm plots, dependence plots, density plots, expression–SHAP scatter plots, waterfall plots, force plots, clustered heatmaps, and cumulative contribution curves were generated to characterize global and sample‐level feature contributions. An 80% cumulative contribution threshold was used as a reference for identifying the principal predictive features. Permutation feature importance was additionally evaluated using DALEX to assess the robustness of the main SHAP‐ranked features.

All machine learning analyses were performed in R 4.5.2. The candidate‐gene modeling workflow used glmnet v4.1–10, caret v7.0–1, mboost v2.9–11, randomForestSRC v3.6.2, gbm v2.2.3, xgboost v3.2.0.1, e1071 v1.7–17, MASS v7.3–65, plsRglm v1.7.0, class v7.3–23, ada v2.0–5.1, pROC v1.19.0.1, and ComplexHeatmap v2.26.1. The SHAP workflow additionally used randomForest, kernlab, klaR, kernelshap, shapviz, and DALEX. Package versions that could not be recovered from the archived analysis environment are explicitly identified in Table S3.

### 2.4 Clinical Relevance and External Validation

Overall survival (OS) was defined as the interval from diagnosis to death or last follow‐up. Patients were stratified into high‐ and low‐expression groups according to the median expression level of each target gene. Kaplan–Meier curves and log‐rank tests were used to compare survival between groups, and univariate Cox proportional hazards regression was performed to estimate hazard ratios (HRs) and corresponding confidence intervals (CIs). Because comprehensive clinical and molecular covariates were not uniformly available across all datasets, these analyses were interpreted as exploratory survival association analyses rather than evidence that the target genes are independent prognostic factors. Survival analyses were performed in R using survival v3.8–3, survminer v0.5.2, ggplot2 v4.0.2, dplyr v1.2.0, and maxstat v0.7–26. The expression patterns and survival associations of the target genes were further examined across cancer types using GEPIA2, including tumor–normal expression comparison and associations with OS or disease‐free survival (DFS).

Protein‐level validation was performed using the Human Protein Atlas (HPA) and the Clinical Proteomic Tumor Analysis Consortium (CPTAC) resource. HPA immunohistochemistry was reviewed as representative and largely qualitative staining evidence according to the official interpretation criteria, whereas CPTAC was used for independent quantitative proteomic assessment. Normalized CPTAC proteomic expression values were retrieved from the CPTAC Shiny platform and used to compare target protein abundance between GBM and control tissues descriptively and/or statistically according to the available data format.

### 2.5 Single‐Cell Transcriptomic Analysis

Single‐cell RNA sequencing data were processed using the Seurat package. Cells were retained according to the following quality‐control criteria: 200–5500 detected genes per cell, at least 600 unique molecular identifiers (UMIs), mitochondrial gene proportion < 5%, and hemoglobin gene proportion < 10%. After quality control, the data were normalized, highly variable genes were identified, and PCA was performed, followed by batch‐effect correction using Harmony. Cell clustering was performed using a shared nearest‐neighbor graph‐based approach, and uniform manifold approximation and projection (UMAP) was used for two‐dimensional visualization. Major cell types were annotated according to canonical marker genes. To distinguish malignant from nonmalignant cells, copy number variation (CNV) profiles were inferred using inferCNV, and cell clusters showing clear chromosomal copy‐number alterations were considered putative malignant tumor cells. These malignant cells were then extracted and re‐clustered to further examine transcriptional heterogeneity within the tumor‐cell compartment. Trajectory analysis was performed using partition‐based graph abstraction (PAGA) and Slingshot: PAGA was first used to estimate connectivity among malignant‐cell states, and Slingshot was then applied to infer lineage relationships and pseudotime ordering. In parallel, genes co‐expressed with the core target genes were identified, and gene set variation analysis (GSVA) was used to estimate the activity of related transcriptional programs across tumor subpopulations and cell states. To explore the predicted network‐level effects of S100B perturbation, in silico knockout analysis was performed using scTenifoldKnk. Regulatory networks before and after virtual knockout were compared topologically, and genes showing significant predicted changes were subjected to functional enrichment analysis. Cell–cell communication analysis was conducted using CellChat, in which ligand–receptor interactions among annotated cell subsets were inferred, and the number and strength of interactions, as well as the dominant signaling pathways, were compared across cell states to identify potential sender and receiver populations within the tumor microenvironment.

### 2.6 Spatial Transcriptomics Analysis

Spatial transcriptomic data were analyzed using a Seurat‐based workflow. Spatial objects containing gene‐expression matrices and tissue‐coordinate information were generated for each sample and subjected to quality assessment. After SCTransform normalization, PCA was performed for dimensionality reduction, followed by graph‐based clustering and UMAP visualization to identify spatial domains. Differentially expressed genes in each spatial cluster were identified using FindAllMarkers, and spatially variable feature analysis was performed to detect genes with nonrandom spatial expression patterns. Spatial feature plots were generated to visualize the in situ distribution of the genes of interest on tissue sections. SingleR was then used with a human reference dataset to infer the predominant cell‐type identity of each spatial spot, and the inferred spot annotations were visualized both in low‐dimensional embedding space and in the original tissue coordinates to assist interpretation of spatial gene‐expression patterns.

### 2.7 Radiogenomic Analysis

Preoperative contrast‐enhanced T1‐weighted imaging (CE‐T1WI) and matched transcriptomic data from 92 patients in publicly available datasets were used for radiogenomic analysis. The radiogenomic cohort was randomly divided into a training set and an internal validation set at a ratio of 7:3. No independent external radiogenomic validation cohort was available in the present study.

Tumor regions of interest (ROIs) were manually delineated on CE‐T1WI by two experienced radiologists who were blinded to clinical information and patient outcomes. Discrepancies in tumor segmentation were reviewed and resolved by consensus. To reduce the influence of interobserver variability, radiomic feature reproducibility was assessed using the intraclass correlation coefficient (ICC), and features meeting the predefined reproducibility criterion were retained for subsequent analysis. Detailed information on cohort eligibility, MRI acquisition parameters, image–transcriptome matching, ROI segmentation, feature reproducibility assessment, and data‐splitting procedures is provided in Table S2.

Radiomic features were extracted from the original and filtered CE‐T1WI images using a standardized PyRadiomics/OnekeyAI‐based workflow. Image preprocessing, radiomic feature categories, feature‐extraction procedures, software resources, and feature reproducibility criteria are summarized in Table S2. Feature preprocessing, feature selection, radiomics‐score construction, model development, and hyperparameter tuning were performed exclusively within the training set to reduce the risk of information leakage. Multilayer perceptron, random forest, and ExtraTrees classifiers were constructed using the selected radiomic features. Model discrimination was evaluated in the internal validation set using receiver operating characteristic curves and the area under the curve. Decision curve analysis was conducted as an exploratory assessment of model‐derived net‐benefit patterns.

Voxel‐wise habitat mapping was performed to explore intratumoral imaging heterogeneity. Voxel‐level radiomic features were calculated from CE‐T1WI using the corresponding tumor segmentation masks and were linearly combined according to the coefficients of the predefined radiogenomic model to generate voxel‐wise habitat score maps. These maps were used to visualize the spatial distribution of imaging‐derived habitats within individual tumors. Radiogenomic modeling and habitat mapping were interpreted as exploratory associations between MRI‐derived phenotypes and gene‐expression characteristics rather than as evidence of causal relationships. Given the absence of an independent external validation cohort, the generalizability of the radiogenomic models requires further evaluation in independent multicenter cohorts.

### 2.8 Statistical Analysis

All statistical analyses were performed in R unless otherwise specified. Continuous variables were analyzed using parametric or nonparametric methods according to data distribution, and categorical variables were compared using chi‐squared or Fisher's exact tests where applicable. Kaplan–Meier analysis and the log‐rank test were used for survival comparison, and univariate Cox proportional hazards regression was used to estimate hazard ratios and confidence intervals. Correlations were evaluated using Spearman correlation analysis. For analyses involving multiple comparisons, *p* values were adjusted using the Benjamini–Hochberg false discovery rate method where applicable. A two‐sided *p* value < 0.05 was considered statistically significant.

Results from ssGSEA, inferCNV, pseudotime inference, CellChat, SingleR annotation, in silico perturbation analysis, spatial transcriptomic annotation, and radiogenomic habitat mapping were interpreted as computational inferences or hypothesis‐generating associations rather than definitive causal evidence. Detailed package versions, algorithm settings, and representative analytical procedures are summarized in Supplementary Table S3.

## 3 Results

### 3.1 Integrated Transcriptomic Analysis Reveals GBM‐Associated Changes in Extracellular Matrix Remodeling, Proliferation, Immune Signaling, and Neural‐Function Programs

**FIGURE 1:**
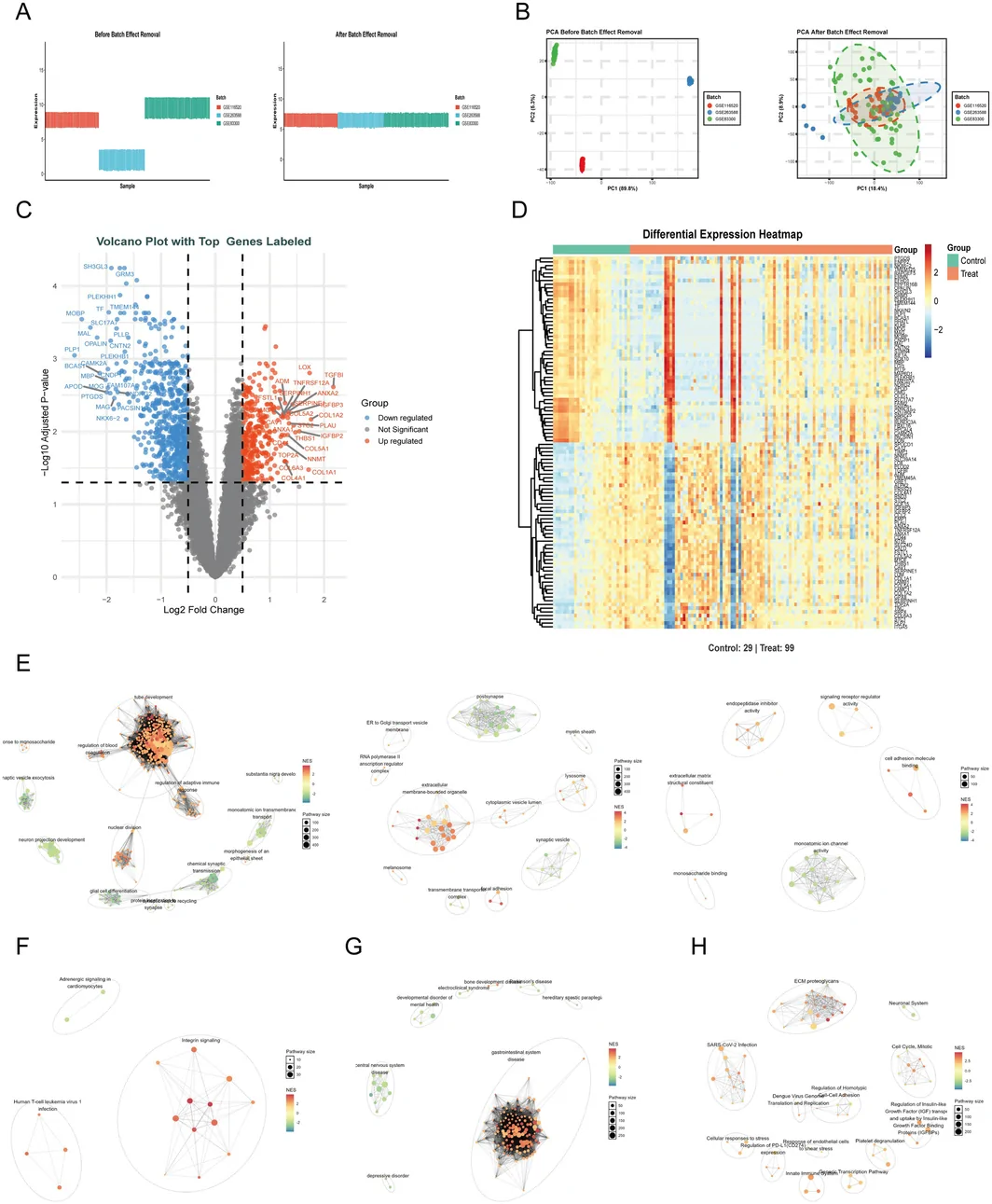
Batch correction, differential expression analysis, and functional enrichment profiling of GBM‐related transcriptomic signatures. (A) Sample expression distributions before and after batch‐effect correction across the integrated datasets. (B) PCA plots before and after batch correction, showing improved integration of samples from different datasets after adjustment. (C) Volcano plot of differentially expressed genes (DEGs) between GBM and control samples. Red dots indicate upregulated genes, blue dots indicate downregulated genes, and gray dots indicate nonsignificant genes. DEGs were defined using |log_2_FC| > 0.5 and adjusted *p* < 0.05. (D) Heatmap of DEGs showing distinct expression patterns between GBM and control samples. Rows represent genes and columns represent samples. (E) GO enrichment network of DEGs, including BP, CC, and MF categories. (F) KEGG pathway enrichment network showing representative enriched pathways among DEGs. (G) DO enrichment network showing disease‐related annotations associated with the DEGs. (H) Reactome pathway enrichment network highlighting major biological pathways associated with the DEGs. In the enrichment networks, node size represents the number of genes in each term or pathway, and node color indicates the normalized enrichment score (NES).

Three independent GBM datasets, GSE116520, GSE263588, and GSE83300, were merged for transcriptomic analysis and biomarker screening. Before batch correction, boxplots showed differences in overall expression distributions among cohorts (Figure 1A), and PCA revealed clear separation according to dataset source (Figure 1B), indicating substantial inter‐cohort variation. Batch effects were therefore adjusted using the removeBatchEffect function in the limma package. After correction, expression distributions became more consistent across datasets (Figure 1A), and PCA showed improved overlap among samples from different cohorts (Figure 1B). The corrected matrix was then used for subsequent analyses. Differential expression analysis between GBM and control samples was performed using |log_2_FC| > 0.5 and adjusted *p* < 0.05 as thresholds. The volcano plot revealed extensive transcriptional differences between the two groups (Figure 1C). Several extracellular matrix‐related genes, including COL1A1, COL1A2, COL6A3, and SERPINH1, were upregulated in GBM samples, consistent with enhanced extracellular matrix organization and stromal remodeling. By contrast, myelin‐ and oligodendrocyte‐associated genes, including MBP, PLP1, and NKX6‐2, were downregulated, reflecting reduced neural and myelin‐related expression programs in GBM. Hierarchical clustering based on differentially expressed genes separated most GBM samples from control samples (Figure 1D).

Functional enrichment analysis further supported these transcriptional changes. GO enrichment showed associations with developmental processes, regulation of coagulation, adaptive immune response, nuclear division, and several nervous system‐related processes, including neuron projection development, chemical synaptic transmission, synaptic vesicle cycling, and ion transmembrane transport (Figure 1E). KEGG and Reactome analyses highlighted integrin signaling, extracellular matrix proteoglycans, cell‐cycle and mitotic pathways, innate immune responses, and vascular or endothelial response programs (Figure 1F,H). Disease Ontology analysis linked the differentially expressed genes to central nervous system disease categories and neurodegeneration‐related terms (Figure 1G). Together, these findings indicate that GBM samples were characterized by upregulation of extracellular matrix remodeling, proliferative activity, immune‐related signaling, and vascular or endothelial programs, whereas neural, synaptic, and myelin‐associated signatures were relatively reduced. These results provided the basis for subsequent microbiota‐associated candidate‐gene screening and biomarker prioritization.

### 3.2 Machine‐Learning Prioritization Supports S100B and ITGB5 as Candidate Signals With Distinct Biological Contexts

**FIGURE 2:**
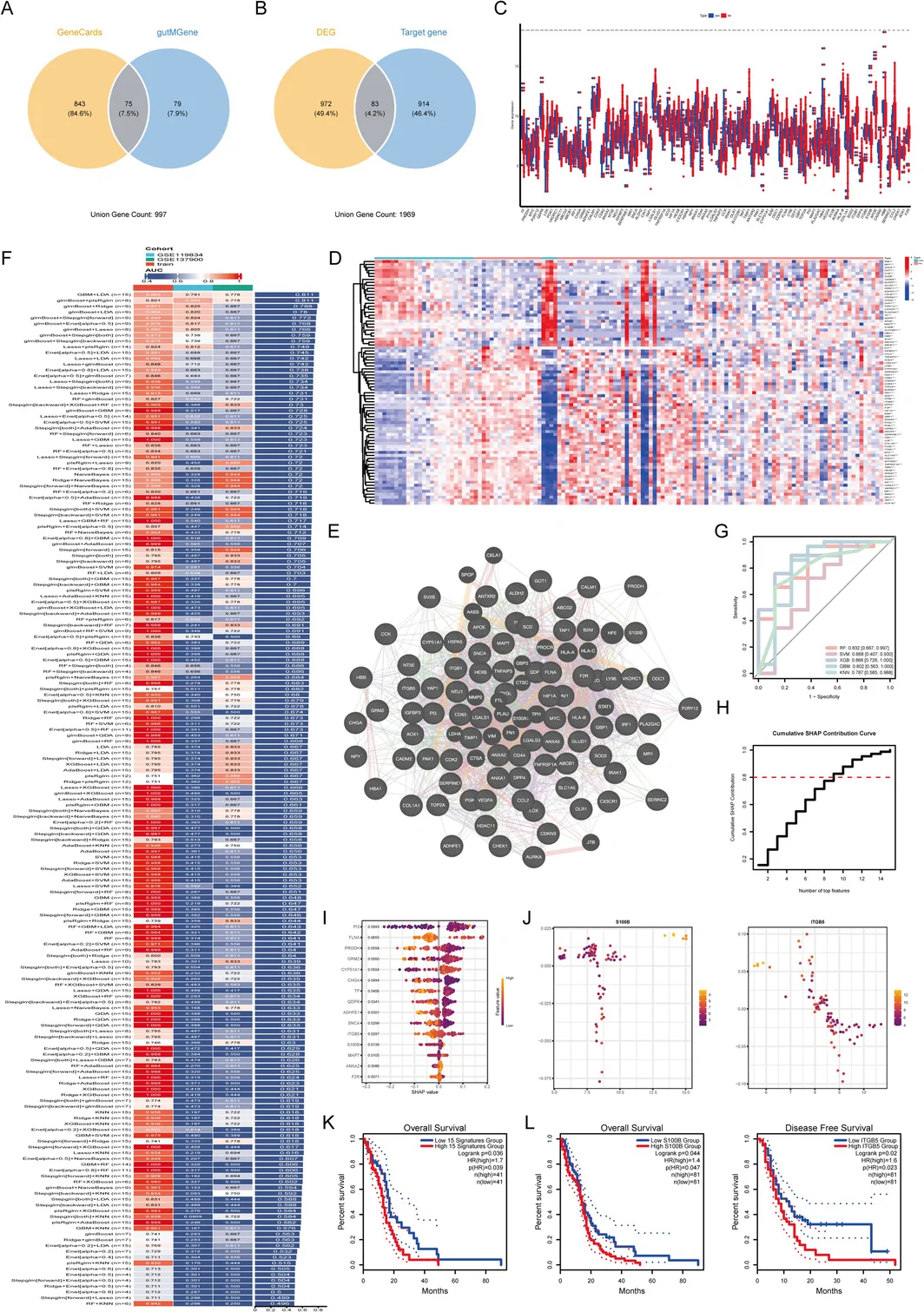
Hypothesis‐generating screening and machine‐learning prioritization of microbiota‐associated candidate genes in GBM. (A) Venn diagram showing the overlap between microbiota‐associated genes retrieved from the GeneCards and gutMGene databases, yielding 997 unique candidate genes, including 75 shared genes. (B) Intersection of the DEGs with the microbiota‐associated candidate genes identified 83 overlapping genes for subsequent analyses. (C) Expression distribution of the 83 overlapping genes across samples, showing marked heterogeneity. (D) Hierarchical clustering heatmap of the 83 candidate genes, demonstrating an overall separation of samples according to disease status. (E) GeneMANIA network analysis of the candidate genes, revealing extensive co‐expression patterns and functional associations. (F) Heatmap of the AUC values for different combinations of feature‐selection methods and classification algorithms, showing variable diagnostic performance across machine‐learning pipelines; the glmBoost + stepglm (forward) model achieved the best overall performance. (G) ROC curves of representative high‐performing models in the training set and independent validation cohorts, including GSE119834 and GSE137900, showing moderate and relatively consistent classification performance across the analyzed cohorts.

Genes related to microbiota‐associated processes were collected from GeneCards and gutMGene as a candidate screening set. After duplicate removal, 997 unique genes were retained, including 75 genes shared by both databases (Figure 2A). Intersecting these genes with the GBM‐related differentially expressed genes identified above yielded 83 overlapping genes for downstream analysis (Figure 2B). The expression patterns of these 83 genes differed between GBM and control samples, with visible inter‐sample variation (Figure 2C), and hierarchical clustering based on these genes separated most GBM samples from controls (Figure 2D). Because these 83 genes were derived from the differential expression results, these visualizations were used to illustrate the screening outcome rather than provide independent validation. In addition, this overlap‐based strategy should be interpreted as identifying microbiota‐associated or microbiota‐relevant candidates within GBM‐related transcriptional changes, not as evidence that the genes were directly regulated by the microbiota in GBM. GeneMANIA analysis further showed co‐expression and functional links among several candidate genes (Figure 2E), which were interpreted as database‐derived associations rather than experimentally verified regulatory interactions.

**FIGURE 3:**
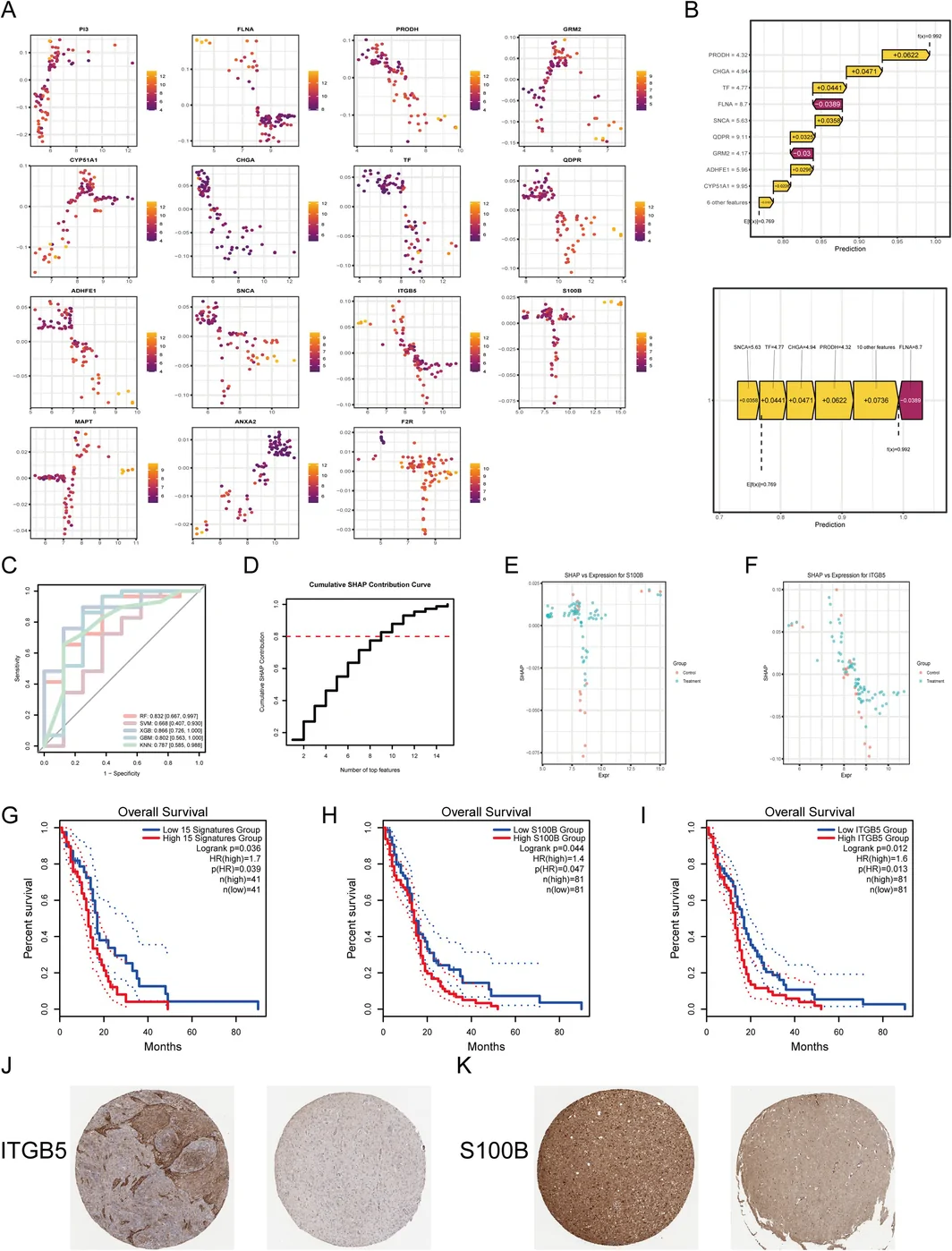
SHAP analysis prioritizes S100B and ITGB5 and evaluates their survival associations in GBM. (A) SHAP dependence plots of the top‐ranked features in the XGBoost model, showing substantial heterogeneity in feature effects and directions across genes. (B) Representative SHAP waterfall and force plots illustrating the positive and negative contributions of major features to model prediction. (C) ROC curves of five classifiers constructed using the selected core gene set, showing that XGBoost achieved the best discriminative performance. (D) Cumulative SHAP contribution curve showing that the top 10 features accounted for more than 80% of the overall predictive contribution. (E, F) SHAP dependence plots for S100B and ITGB5, respectively, demonstrating clear expression–effect relationships and supporting their substantial contributions to model discrimination. (G) Kaplan–Meier overall survival analysis based on the composite 15‐gene signature score. (H, I) Kaplan–Meier overall survival curves for S100B and ITGB5 in the TCGA‐GBM cohort, showing that higher expression of either gene was associated with shorter overall survival in the analyzed cohort. (J, K) Representative Human Protein Atlas immunohistochemical images illustrating detectable ITGB5 and S100B staining in GBM and normal brain tissues. These images are presented as qualitative staining examples and were not used for quantitative tumor–normal comparison.

Different combinations of feature‐selection methods and classification algorithms were then tested to evaluate the diagnostic‐model contribution of the 83 candidate genes. The AUC heatmap showed clear performance differences among model pipelines (Figure 2F). The glmBoost + stepglm (forward) model showed the best overall performance, with an average AUC of approximately 0.772, while glmBoost + stepglm (alpha = 0.5) and glmBoost + Lasso also performed relatively well. ROC analysis showed that several top‐ranked models maintained moderate classification performance in both the training set and the independent validation cohorts GSE119834 and GSE137900 (Figure 2G). Based on these machine‐learning results, S100B and ITGB5 were retained for further validation and biological interpretation, reducing the initial microbiota‐associated candidate list to two genes with potential diagnostic‐model relevance in GBM. Importantly, these genes were not interpreted only as markers selected from a computational pipeline; they were subsequently assessed as two candidate signals that may represent different dimensions of GBM ecology. For model interpretation, five commonly used classifiers were further trained using the selected core gene set. Among them, XGBoost showed the highest discrimination ability, with an AUC of 0.866 (95% CI: 0.726–1.000), followed by random forest (AUC = 0.832) and the gradient boosting machine classifier (AUC = 0.802) (Figure 3C). XGBoost was therefore selected for subsequent SHAP‐based interpretation.

SHAP analysis was performed to examine the contribution of individual genes to model prediction. Dependence plots showed variable contributions from several genes, including PI3, FLNA, PRODH, GRM2, CYP51A1, CHGA, TF, QDPR, ADHFE1, SNCA, ITGB5, S100B, MAPT, ANXA2, and F2R (Figure 3A). The waterfall plot indicated that individual predictions depended on the combined effects of multiple genes rather than a single dominant feature (Figure 3B). SHAP results further revealed heterogeneity in both the direction and strength of feature effects, while cumulative SHAP analysis showed that the top 10 features contributed more than 80% of the total predictive information (Figure 3D), suggesting that model discrimination was mainly driven by a limited subset of genes. Among these features, S100B and ITGB5 showed expression‐dependent SHAP patterns (Figure 3E,F). ITGB5 was associated with lower SHAP values within specific expression ranges, whereas S100B showed a clearer shift in SHAP values across certain expression intervals. Finally, when diagnostic‐model contribution was considered together with the exploratory survival analyses, S100B and ITGB5 were the only genes showing both model‐level predictive contributions and statistically significant associations with overall survival in the analyzed cohort. They were therefore prioritized for further multi‐omics characterization.

### 3.3 S100B And ITGB5 Are Survival‐Associated Candidate Markers in GBM, With Stronger Quantitative Protein‐Level Support for ITGB5

After prioritizing S100B and ITGB5 as candidate genes, we examined their associations with clinical outcome and protein expression. In the TCGA‐GBM cohort, patients with higher expression of either gene had shorter overall survival, with significant associations observed for both S100B (*p* = 0.044, HR = 1.4) and ITGB5 (*p* = 0.012, HR = 1.6) (Figure 3H,I). A composite score based on the top‐ranked candidate genes also separated patients into groups with different survival outcomes (Figure 3G). Because both genes contributed to the diagnostic models and were associated with overall survival in the analyzed cohort, they were selected for further characterization. These univariate analyses do not establish either gene as an independent prognostic factor.

**FIGURE 4:**
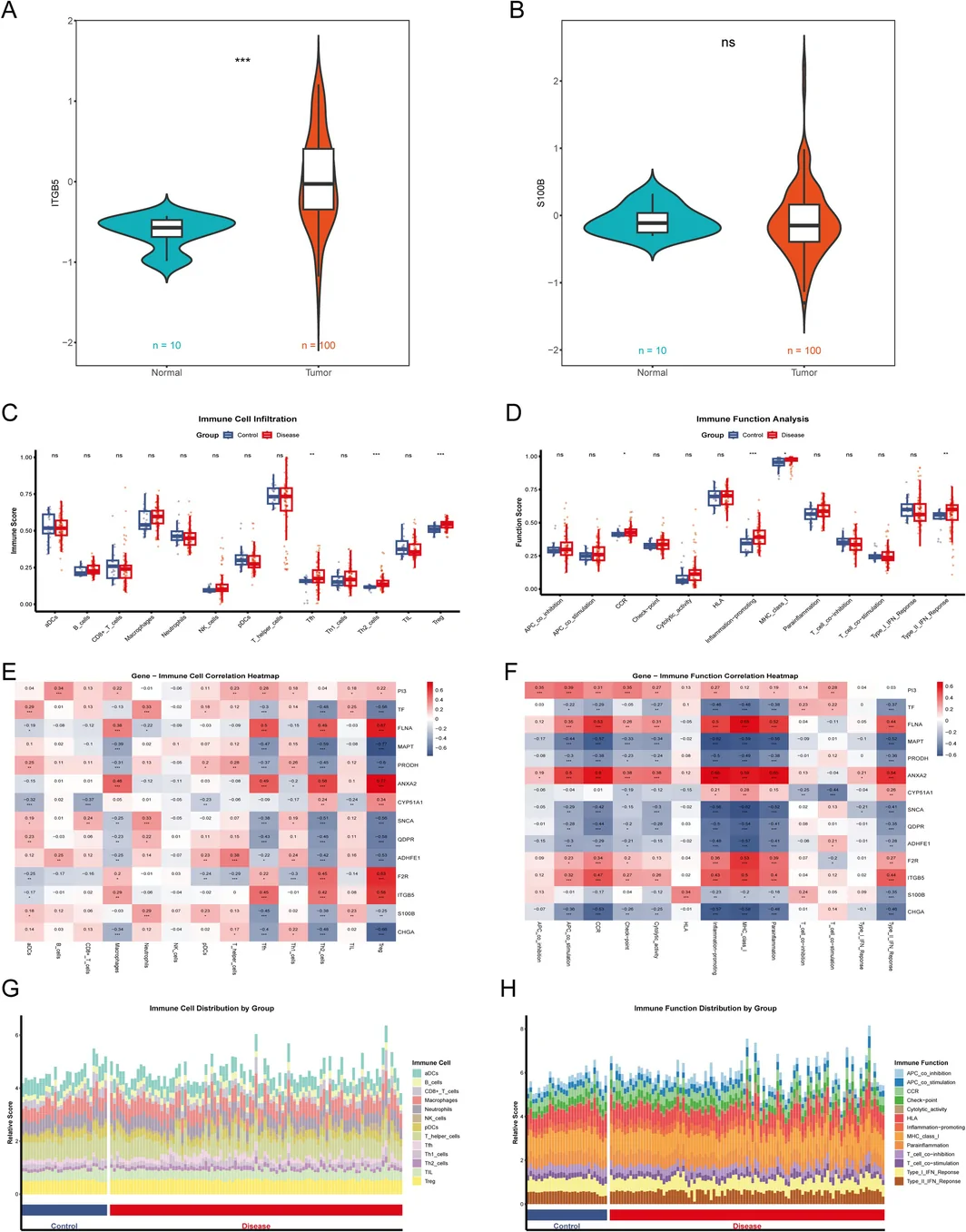
Protein‐level support and bulk immune microenvironment associations of S100B and ITGB5 in GBM. (A) Violin plot comparing ITGB5 expression between normal brain tissues and GBM tissues in the CPTAC cohort. (B) Violin plot comparing S100B expression between normal brain tissues and GBM tissues in the CPTAC cohort. (C) ssGSEA‐based comparison of immune‐cell infiltration between control and GBM groups, showing differences in the relative abundance of major immune cell populations. (D) Comparison of immune‐related functional signatures between control and GBM groups, including antigen presentation, inflammatory response, checkpoint activity, and T‐cell regulatory programs. (E) Correlation heatmap showing the associations between candidate‐gene expression and major infiltrating immune‐cell populations. (F) Correlation heatmap showing the associations between candidate‐gene expression and immune functional signatures. (G) Relative distribution of immune‐cell infiltration patterns across samples in the control and GBM groups. (H) Relative distribution of immune functional patterns across samples in the control and GBM groups.

Protein‐level evidence was first assessed using immunohistochemical images from the Human Protein Atlas (HPA). Representative images showed detectable staining for both ITGB5 and S100B in GBM tissues (Figure 3J,K). ITGB5 showed moderate to strong staining in tumor tissue, whereas S100B showed broader and more intense staining in the displayed GBM samples. Because the HPA results were based on representative staining images, they were treated as qualitative evidence rather than quantitative validation. Quantitative proteomic assessment was then performed using CPTAC data. ITGB5 protein abundance was significantly higher in GBM tissues than in normal controls (*p* < 0.001; Figure 4A), consistent with the transcriptomic results. By contrast, S100B did not show a significant tumor‐normal difference in CPTAC (*p* > 0.05; Figure 4B), although inter‐sample variability was apparent. This inconsistency may be related to cohort composition, post‐transcriptional regulation, tissue context, or tumor heterogeneity.

Together, these findings indicate that protein‐level support was more consistent for ITGB5 than for S100B. ITGB5 was supported by transcriptomic upregulation, survival association, immunohistochemical staining, and CPTAC proteomic validation. S100B, in contrast, was supported mainly by diagnostic model contribution, survival association, and qualitative immunohistochemical evidence, but not by significant differential protein abundance in CPTAC. Pan‐cancer analysis further showed broader tumor‐associated expression of ITGB5, whereas S100B displayed a more tissue‐ or lineage‐dependent expression pattern. Detailed pan‐cancer expression and survival‐association results are provided in Figure S1. Because S100B and ITGB5 contributed to the diagnostic models and were associated with overall survival in the TCGA‐GBM cohort, they were selected for further characterization.

### 3.4 S100B And ITGB5 Are Associated With Distinct Immune‐Related Patterns in GBM

Because bulk transcriptomic analysis showed enrichment of immune‐ and stroma‐related programs in GBM, we examined the associations of S100B and ITGB5 with tumor immune features using ssGSEA‐based immune‐cell and immune‐function signatures. At the immune‐cell level, several signatures differed between GBM and control samples (Figure 4C). Tfh, Th2, and Treg scores were significantly higher in GBM samples, although not all immune‐cell populations followed the same pattern. These findings suggest a selective change in the GBM immune landscape rather than a uniform increase in immune infiltration.

Correlation analysis revealed distinct immune‐association patterns for S100B and ITGB5 (Figure 4E). ITGB5 was positively correlated with several immune‐cell signatures, including Treg and macrophage‐related scores, consistent with a possible association between ITGB5 and an immunoregulatory tumor microenvironment. In contrast, S100B showed a less uniform pattern, with negative correlations with Treg, Tfh, and Th2 signatures and variable associations with other immune‐cell subsets. These results indicate that ITGB5 may be more consistently linked to immune‐cell infiltration or immune regulation, whereas the immune‐related associations of S100B appear more heterogeneous.

Immune‐function analysis showed that GBM samples had higher scores for CCR, inflammation‐promoting activity, MHC Class I, and type II IFN response (Figure 4D), indicating that the GBM immune context contains both inflammatory and immunoregulatory features rather than fitting a simple immune‐desert pattern. Consistent with the immune‐cell results, ITGB5 was positively correlated with multiple immune‐function signatures, including inflammation‐promoting activity, checkpoint‐related signaling, and MHC Class I‐related features (Figure 4F), whereas S100B again showed mixed associations with immune‐function scores. The distribution of immune‐cell and immune‐function signatures across individual samples is also shown (Figure 4G,H). Overall, ITGB5 showed a more consistent relationship with inflammatory and immunoregulatory signatures in GBM, while S100B showed a more heterogeneous immune‐correlation pattern, suggesting that its immune‐related associations may depend more strongly on cell state or local tumor context.

### 3.5 Single‐Cell Transcriptomic Analysis Shows Different Cellular Distributions of S100B and ITGB5 in GBM

**FIGURE 5:**
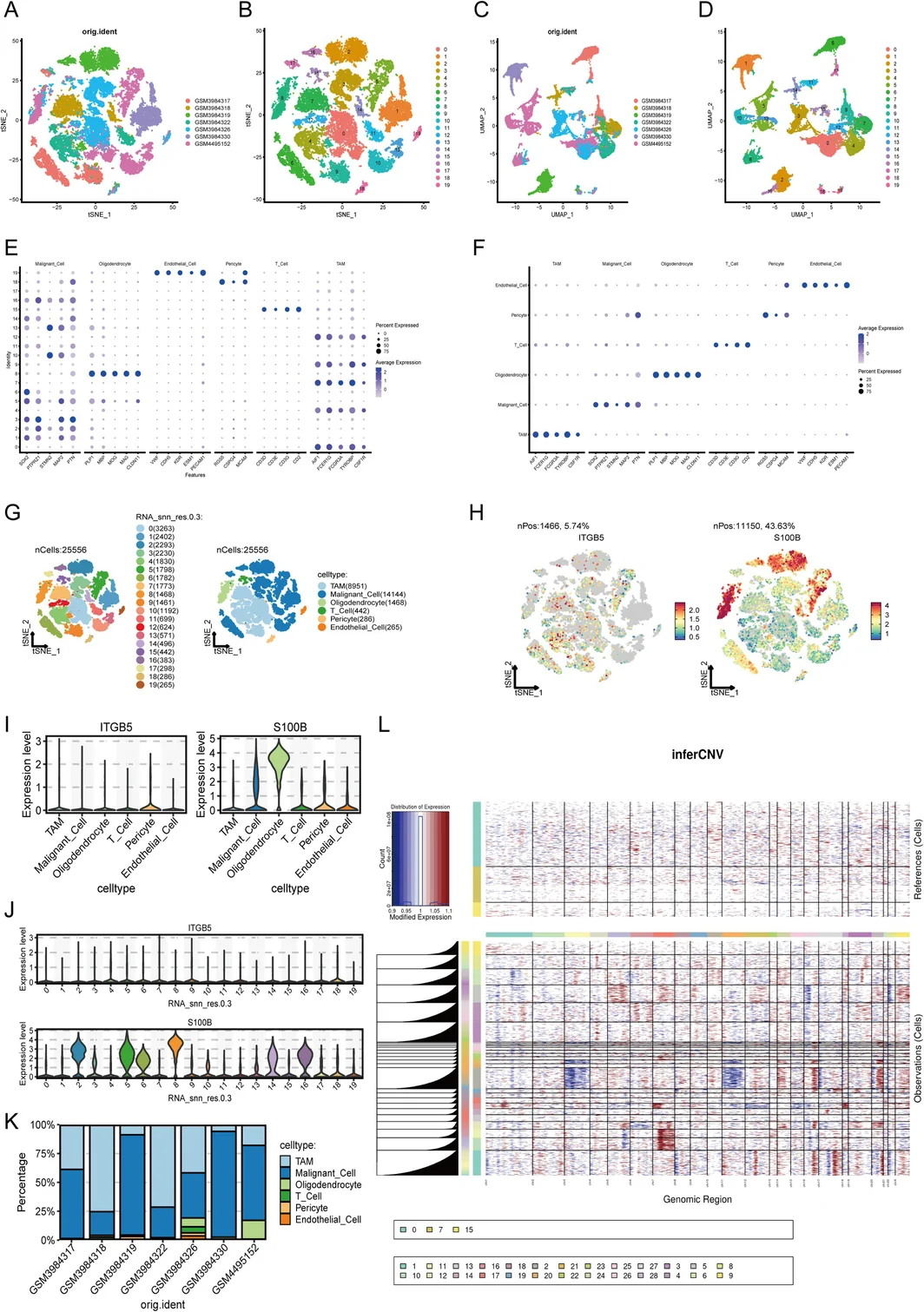
Single‐cell transcriptomic analysis reveals cellular composition, target‐gene distribution, and inferred copy‐number heterogeneity in the GBM microenvironment. (A) t‐SNE plot of the GSE135045 single‐cell dataset colored by sample origin. (B) t‐SNE plot colored by unsupervised cluster assignment, showing 20 identified clusters. (C) UMAP plot of the GSE135045 single‐cell dataset colored by sample origin. (D) UMAP plot colored by cluster assignment. (E, F) Dot plots showing the expression patterns of canonical marker genes across major cell populations, used for cell‐type annotation. (G) Annotated low‐dimensional embedding showing the distribution of major cell types, including malignant cells, TAMs, oligodendrocytes, T cells, pericytes, and endothelial cells. (H) Feature plots showing the single‐cell expression distributions of ITGB5 and S100B, together with the proportions of positive cells. (I) Violin plots showing ITGB5 and S100B expression across annotated cell types. (J) Violin plots showing ITGB5 and S100B expression across individual clusters. (K) Stacked bar plot showing the relative proportions of major cell types across different samples. (L) inferCNV heatmap showing large‐scale copy‐number variation patterns in malignant cells relative to nonmalignant reference cells.

To examine the cellular context of S100B and ITGB5, we analyzed integrated single‐cell RNA‐seq data from GBM samples. Quality‐control analysis showed a strong correlation between sequencing depth (nCount_RNA) and the number of detected genes (nFeature_RNA) (*r* = 0.94), whereas mitochondrial gene proportion was not clearly related to sequencing depth (Figure S2A–C). After normalization, the top 2000 highly variable genes were selected for dimensionality reduction and clustering, and clustree analysis was used to assist the selection of clustering resolution (Figure S2D,E). The integrated single‐cell atlas was divided into multiple clusters and annotated into six major cell populations, including malignant cells marked by SOX2 and PTPRZ1, oligodendrocytes marked by PLP1 and MBP, endothelial cells, pericytes, T cells, and tumor‐associated macrophages marked by AIF1 (Figure 5A–G).

Mapping the expression of S100B and ITGB5 revealed distinct distribution patterns across the GBM cellular ecosystem (Figure 5H–K). S100B was broadly expressed across glial‐lineage and tumor‐associated cell populations and was detected in approximately 43.63% of all cells. In contrast, ITGB5 showed a more restricted pattern and was detected in 5.74% of cells, mainly in selected malignant‐cell and microenvironment‐associated subpopulations. These results indicate that S100B represents a broader cellular signal, whereas ITGB5 is more limited to specific cell states or local microenvironment‐related compartments. To support malignant‐cell identification, CNV profiles were inferred using inferCNV. Malignant‐cell populations showed broad CNV‐like alterations compared with nonmalignant reference cells, whereas microenvironmental cells had relatively stable inferred copy‐number profiles (Figure 5L). Different malignant‐cell clusters also showed variable CNV‐like patterns, consistent with intratumoral heterogeneity at the single‐cell level.

**FIGURE 6:**
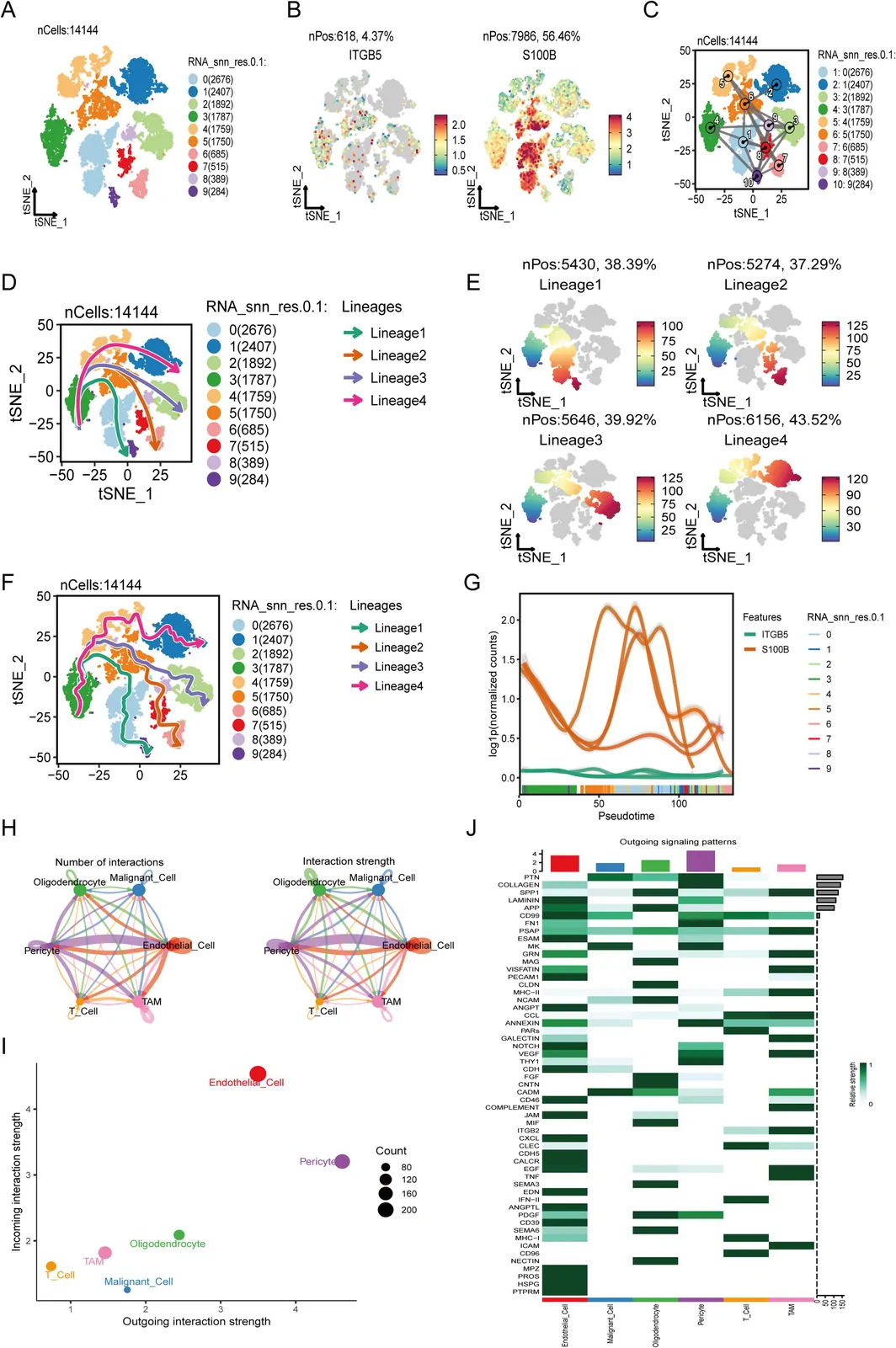
Malignant‐cell subclustering, trajectory inference, and intercellular communication analysis in the GSE135045 single‐cell dataset. (A) t‐SNE plot of the 14,144 putative malignant cells extracted from GSE135045, showing 10 malignant‐cell subclusters. (B) Feature plots showing the expression distributions of ITGB5 and S100B across the re‐clustered malignant‐cell subpopulations, together with the proportions of positive cells. (C) Trajectory inference map showing the pseudotime structure of the re‐clustered malignant‐cell population. (D) Distribution of malignant cells across four major inferred trajectory branches, together with the corresponding branch‐associated cell proportions. (E) Visualization of lineage trajectories in low‐dimensional space, illustrating the major differentiation/state‐transition directions. (F) Lineage‐specific trajectory visualization across the inferred branches. (G) Dynamic expression curves of ITGB5 and S100B along pseudotime. (H) Global intercellular communication networks showing the number of interactions (left) and interaction strength (right) among major cell populations. (I) Scatter plot summarizing outgoing and incoming communication strength for each cell type. (J) Heatmap of outgoing signaling patterns across cell types, showing the relative activity of major ligand–receptor signaling pathways in different cellular compartments.

A total of 14,144 malignant cells were then extracted for further subclustering and trajectory analysis. Re‐clustering identified 10 malignant‐cell subclusters with distinct transcriptional profiles (Figure 6A,B). Pseudotime analysis resolved four major inferred lineage branches within the malignant‐cell compartment (Figure 6C–F). Along these trajectories, S100B showed dynamic expression changes and was more evident from intermediate to later pseudotime states in several branches, whereas ITGB5 showed a narrower distribution, with increased expression mainly in specific branch regions (Figure 6G). Because pseudotime analysis is inferential, these patterns were interpreted as associations with malignant‐cell state variation rather than direct evidence that either gene drives a lineage transition. Overall, the single‐cell analysis showed that S100B and ITGB5 occupied different cellular contexts in GBM. S100B was broadly distributed across glial‐lineage and tumor‐associated states and varied along inferred malignant‐cell trajectories, while ITGB5 was concentrated in selected malignant subpopulations and appeared to be more closely related to restricted cell‐state or niche‐associated programs.

### 3.6 Cell–Cell Communication and S100B Perturbation Analyses Suggest Associations Between S100B‐Related States and Neuroglial, Immune‐Phagocytic, and Metabolic Programs

**FIGURE 7:**
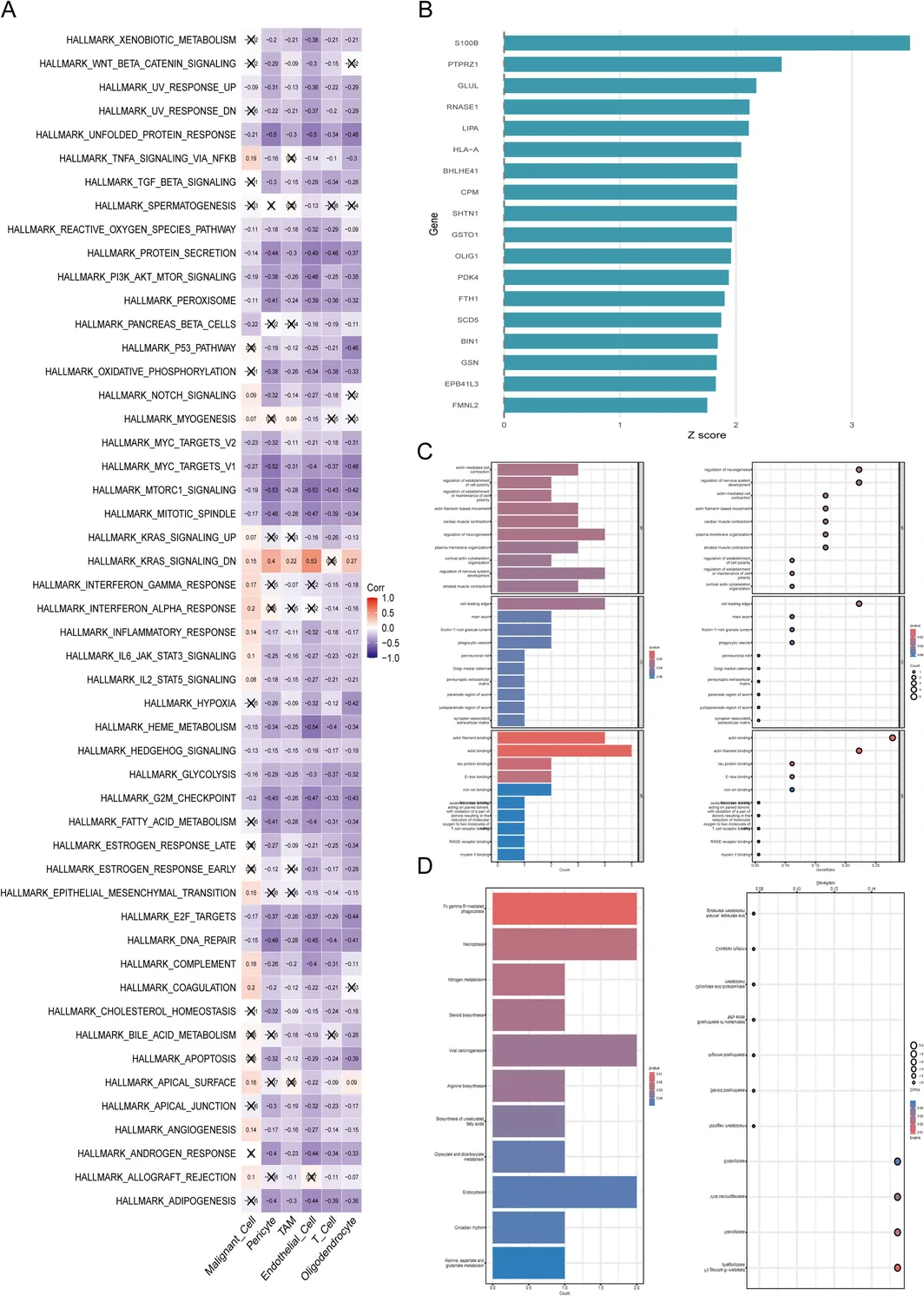
GSVA‐based pathway context and in silico S100B perturbation analysis in GBM. (A) GSVA‐based Hallmark pathway activity landscape associated with ITGB5 and S100B across major GBM‐associated cell populations before S100B perturbation. (B) Ranked perturbation plot showing representative downstream genes most affected by in silico knockout of S100B. (C) Gene Ontology (GO) enrichment analysis of genes associated with S100B perturbation, including biological process (BP), cellular component (CC), and molecular function (MF) categories. (D) KEGG pathway enrichment analysis of genes associated with S100B perturbation.

We next analyzed inferred ligand–receptor interactions among the major cell populations identified in the single‐cell dataset. CellChat analysis showed extensive communication among malignant cells, tumor‐associated macrophages (TAMs), endothelial cells, pericytes, oligodendrocytes, and T cells (Figure 6H,I). Endothelial cells, pericytes, and TAMs showed relatively high inferred communication activity, while malignant cells also showed broad predicted interactions with stromal and immune cell populations. Outgoing signaling‐pattern analysis identified several pathways related to neuroglial signaling, extracellular matrix organization, cell adhesion, vascular communication, and immune recognition, including PTN, COLLAGEN, SPP1, LAMININ, FN1, MHC‐I, MHC‐II, NOTCH, VEGF, PDGF, ICAM, and HSPG‐related pathways (Figure 6J). Before perturbation analysis, GSVA was performed to compare baseline pathway activity across major GBM‐associated cell populations. The GSVA heatmap showed cell‐type‐specific enrichment of Hallmark pathways (Figure 7A), with malignant cells and microenvironmental populations displaying distinct pathway‐activity patterns that provided a reference for interpreting the subsequent S100B perturbation results.

To explore the predicted network‐level changes associated with S100B perturbation, we performed an in silico knockout analysis. The analysis identified a set of genes showing prominent predicted changes following virtual S100B perturbation (Figure 7B). Several glial‐lineage or tumor‐state‐associated genes, including PTPRZ1 and OLIG1, were also ranked highly, together with genes related to antigen presentation, lipid metabolism, oxidative stress, and cytoskeletal organization, including HLA‐A, LIPA, PDK4, FTH1, GSN, EPB41L3, and FMNL2. These results indicate that the S100B‐associated transcriptional network may involve glial‐lineage identity, tumor‐cell state programs, immune‐related features, and cytoskeletal remodeling. Functional enrichment analysis of S100B perturbation‐associated genes further showed enrichment of GO terms related to regulation of neurogenesis, nervous system development, actin‐mediated cell contraction, actin filament‐based movement, plasma membrane organization, cell polarity establishment, leading‐edge formation, and axon‐ or synapse‐associated structures (Figure 7C). KEGG analysis highlighted FcγR‐mediated phagocytosis, endocytosis, necroptosis, and several metabolic pathways, including alanine, aspartate and glutamate metabolism, arginine biosynthesis, steroid biosynthesis, and unsaturated fatty acid biosynthesis (Figure 7D).

We then compared the S100B perturbation‐associated genes with the inferred communication pathways. Several highly ranked genes could be placed within communication contexts identified by CellChat. For example, PTPRZ1 was related to the PTN signaling context, whereas HLA‐A linked the S100B‐associated transcriptional state to MHC‐I‐mediated immune recognition. Cytoskeleton‐ and membrane‐associated genes such as GSN and FMNL2 may reflect cellular response programs connected with microenvironmental signaling. Overall, baseline GSVA, in silico S100B perturbation, enrichment analysis, and CellChat‐based communication analysis suggested that S100B is associated with more than a glial‐lineage expression pattern. Its related transcriptional programs involve malignant‐cell state variation, cytoskeletal and membrane remodeling, immune‐phagocytic processes, and metabolic adaptation and may be embedded in neuroglial, immune, stromal, and vascular communication contexts in GBM. Because these results were derived from computational inference and did not include experimental S100B knockdown or direct before‐and‐after communication analysis, they should be interpreted as hypothesis‐generating associations rather than causal mechanisms.

### 3.7 Spatial Transcriptomics Shows Different in Situ Expression Patterns of S100B and ITGB5

**FIGURE 8:**
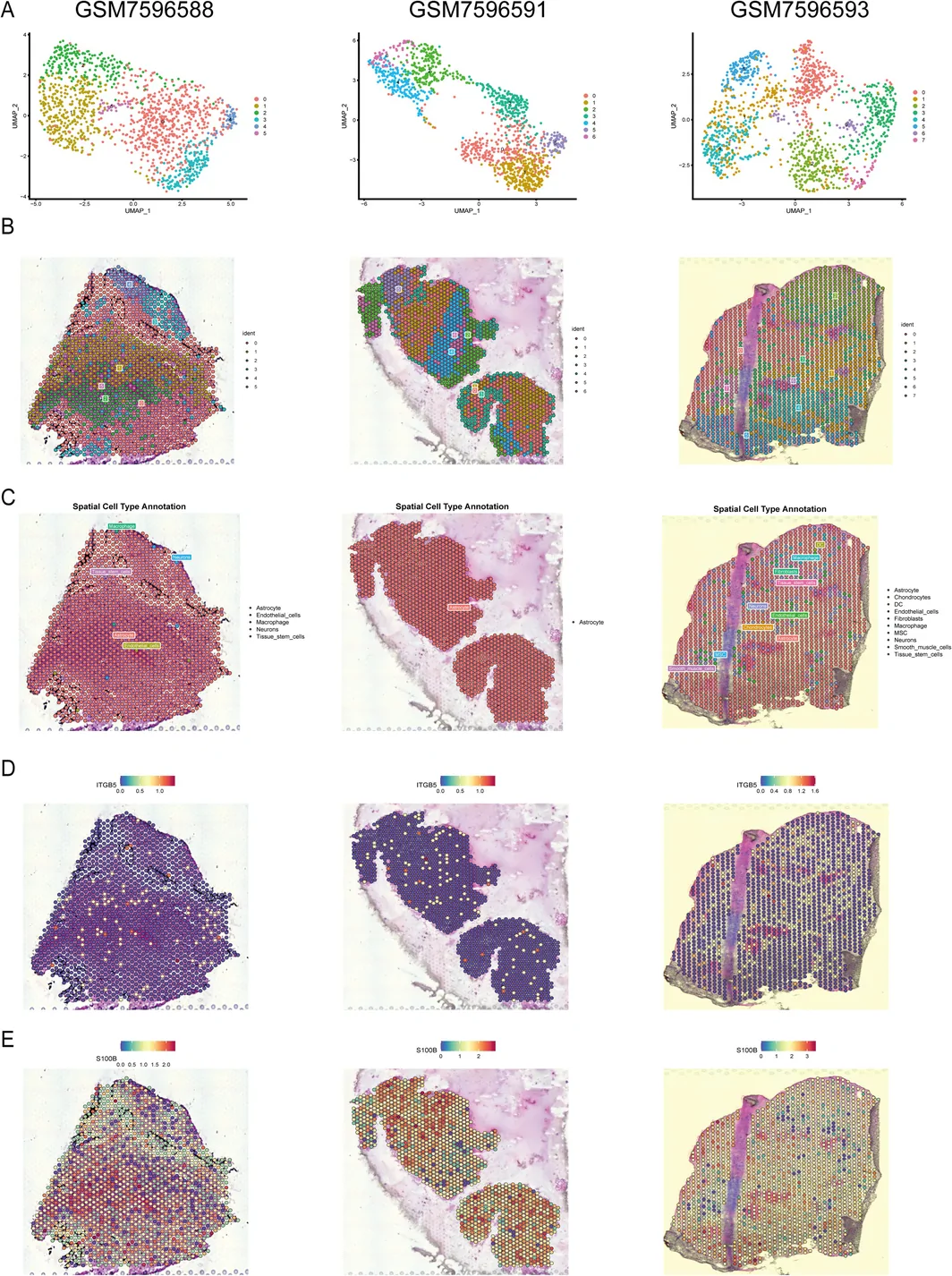
Spatial transcriptomic analysis of S100B and ITGB5 expression in GBM tissues. (A) UMAP visualization of spatial transcriptomic spot clusters in three GBM samples, GSM7596588, GSM7596591, and GSM7596593. (B) Spatial mapping of transcriptional spot clusters onto the corresponding histological sections. (C) Spot‐level cell‐type annotation showing sample‐specific spatial organization of inferred cell populations. (D) Spatial expression patterns of ITGB5 across the three GBM tissue sections. (E) Spatial expression patterns of S100B across the three GBM tissue sections.

Spatial transcriptomic data from three GBM samples, GSM7596588, GSM7596591, and GSM7596593, were analyzed to examine the tissue‐level distribution of S100B and ITGB5. After quality control and SCTransform normalization, PCA, graph‐based clustering, and UMAP analysis identified several spatial spot clusters in each sample (Figure 8A). When projected back onto the tissue sections, these clusters showed nonrandom spatial organization (Figure 8B), indicating spatial heterogeneity within the analyzed GBM tissues. Spot‐level annotation further revealed different spatial patterns among the three samples (Figure 8C). GSM7596588 contained astrocyte‐like, endothelial‐like, macrophage‐like, neuron‐like, and tissue stem cell‐like spots. GSM7596591 was mainly annotated as astrocyte‐like across most spots, suggesting a more uniform glial‐like spatial pattern in this specimen. GSM7596593 showed a more mixed annotation profile, including astrocyte‐like, endothelial‐like, fibroblast‐like, macrophage‐like, neuron‐like, smooth muscle‐like, tissue stem cell‐like, and other stromal or immune‐associated labels. These results indicate clear inter‐sample differences in spatial organization. Because the annotations were inferred at the spot level, they were used to guide interpretation rather than to define exact cellular composition.

Spatial mapping of ITGB5 and S100B showed distinct tissue‐level expression patterns. ITGB5 showed relatively low and restricted expression across the three samples, with signals mainly detected in scattered spots or localized tissue regions (Figure 8D). This distribution was consistent with the single‐cell result showing ITGB5 expression in a limited subset of cells and suggested that ITGB5 may be related to selected malignant or microenvironment‐associated spatial regions. By contrast, S100B showed broader and more diffuse expression across the tissue sections (Figure 8E), with S100B‐positive spots more widely distributed than ITGB5‐positive spots in the analyzed samples. This pattern was consistent with the single‐cell observation that S100B was detected across broader glial lineage and tumor‐associated cell states.

Overall, spatial transcriptomic analysis showed different in situ expression patterns for S100B and ITGB5 in GBM. S100B was more broadly distributed across glial‐ or tumor‐associated spatial regions, whereas ITGB5 showed a more focal pattern. These findings supported the single‐cell results at the tissue level, although they remain descriptive and require experimental validation to clarify the functional roles of these genes.

### 3.8 Radiogenomic Analysis Shows Different Imaging Associations for S100B and ITGB5

**FIGURE 9:**
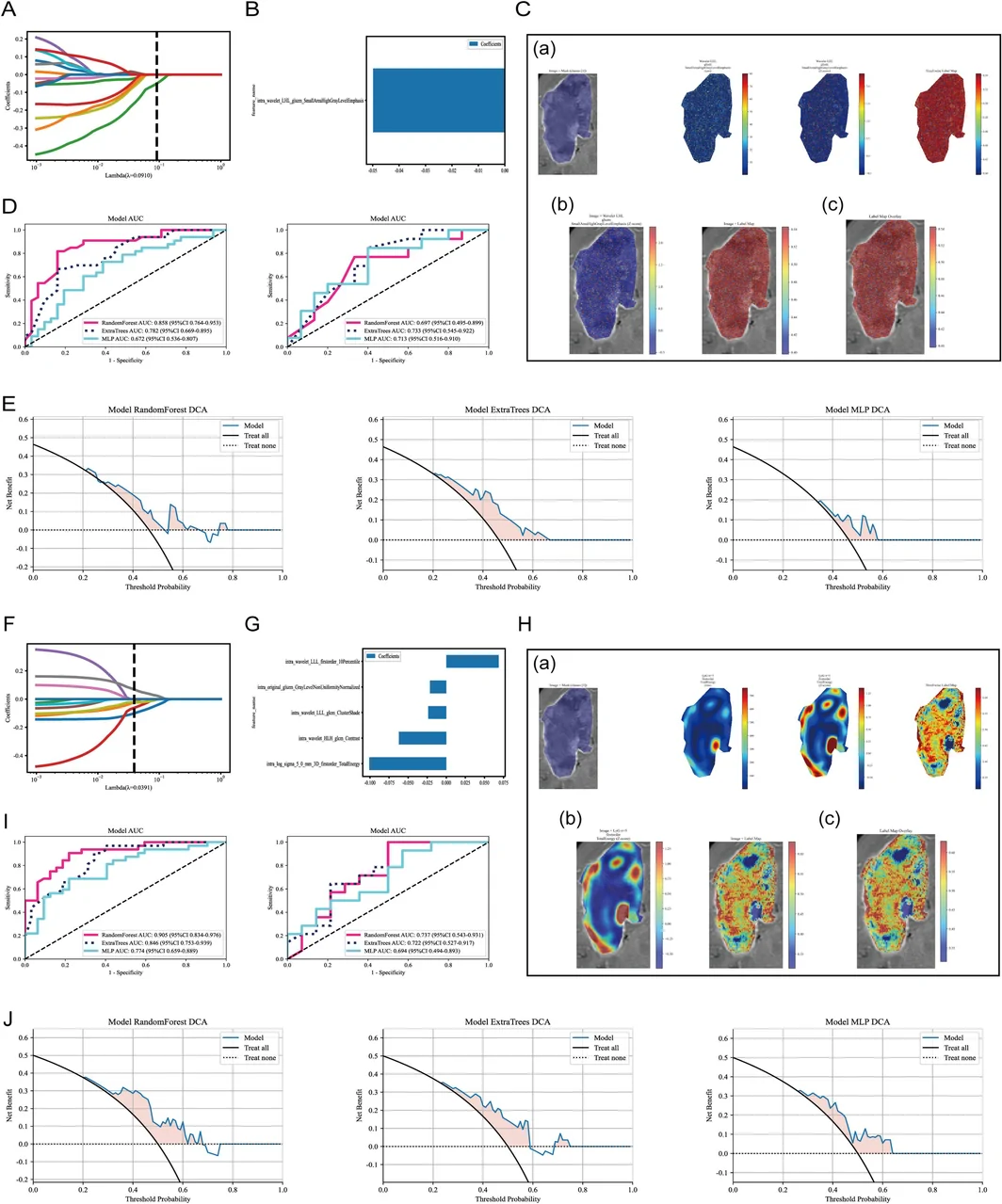
Feature selection, feature visualization, model performance, and decision curve analysis of the S100B‐ and ITGB5‐related radiomics models. (A–E) S100B‐related radiomics model. (A) LASSO coefficient profiles showing progressive shrinkage of most candidate features with increasing penalty; (B) cross‐validation curve for selection of the optimal penalty parameter; (C) coefficient and feature‐map visualization of the retained feature, intra_wavelet_LHL_glszm_SmallAreaHighGrayLevelEmphasis; (D) ROC curves of the random forest, ExtraTrees, and MLP classifiers in the training set and internal validation set; and (E) decision curve analysis showing the net benefit of the model across a range of threshold probabilities compared with the treat‐all and treat‐none strategies. (F–J) ITGB5‐related radiomics model. (F) LASSO coefficient profiles; (G) cross‐validation curve for optimal penalty selection; (H) visualization of the five retained radiomic features, including intra_wavelet_LLL_firstorder_10Percentile, intra_original_glszm_GrayLevelNonUniformityNormalized, intra_wavelet_LLL_glcm_ClusterShade, intra_wavelet_HLH_glcm_Contrast, and intra_log_sigma_5_0_mm_3D_firstorder_TotalEnergy; (I) ROC curves of different classifiers in the training set (left) and internal validation set (right); and (J) decision curve analysis showing exploratory model‐derived net‐benefit patterns across a range of threshold probabilities.

We examined whether S100B and ITGB5 expression were associated with noninvasive imaging features using contrast‐enhanced T1‐weighted imaging (CE‐T1WI)‐based radiogenomic modeling and voxel‐wise habitat mapping. For the S100B‐related model, LASSO feature selection retained one nonzero radiomic feature, intra_wavelet_LHL_glszm_SmallAreaHighGrayLevelEmphasis (Figure 9A,B), which is related to small areas with high gray‐level intensity. This result indicated that the S100B‐related radiomic model was mainly driven by a limited imaging feature. In contrast, for the ITGB5‐related model, LASSO retained five non‐zero radiomic features (Figure 9F,G), which were related to gray‐level distribution, texture nonuniformity, local contrast, and imaging heterogeneity. Compared with the S100B model, the ITGB5‐related model involved a broader set of radiomic features. This difference may be consistent with a more spatially variable or microenvironment‐related imaging phenotype associated with the ITGB5‐related model, although it should not be interpreted as proof that ITGB5 directly determines radiologic heterogeneity.

Voxel‐wise habitat mapping showed a relatively simple intratumoral pattern for the S100B‐related model (Figure 9C), with limited spatial variation within the tumor region. In contrast, the ITGB5‐related habitat map showed a more heterogeneous intratumoral distribution, with localized regions of higher contrast and texture variation (Figure 9H). These findings were consistent with the feature‐selection results and suggested that the ITGB5‐related model showed a more spatially heterogeneous imaging pattern in the analyzed cohort.

We then compared three classifiers, including random forest, ExtraTrees, and multilayer perceptron (MLP), for both radiogenomic models. For the S100B‐related model, the random forest classifier achieved an AUC of 0.858 in the training set and 0.697 in the internal validation set (Figure 9D). For the ITGB5‐related model, the random forest classifier achieved an AUC of 0.905 in the training set and 0.737 in the internal validation set (Figure 9I), indicating slightly better internal validation performance than the S100B‐related model. Decision curve analysis showed that both models provided higher net benefit than the “treat‐all” and “treat‐none” strategies across a range of threshold probabilities (Figure 9E,J). Overall, S100B and ITGB5 showed different MRI‐derived radiomic associations. The ITGB5‐related model involved more radiomic features, showed a more heterogeneous habitat pattern, and had moderately better performance in the internal validation set. Because the internal validation AUCs remained moderate and the performance gap between the training and internal validation sets suggested possible overfitting, these radiogenomic findings should be considered exploratory and require independent multicenter validation before any clinical interpretation or application.

## 4 Discussion

GBM remains one of the most lethal malignancies of the central nervous system, and its clinical aggressiveness is closely related to profound intratumoral heterogeneity, diffuse invasion, and a highly refractory immunosuppressive microenvironment. In recent years, GBM biology has increasingly been viewed beyond a purely tumor‐intrinsic framework, as microbiota‐associated systemic regulation may influence neuroinflammation, metabolic homeostasis, and antitumor immunity, thereby providing a broader context for understanding how local tumor ecosystems are shaped. However, the molecular links between microbiota‐associated signals and remodeling of the GBM microenvironment remain insufficiently defined. In the present study, we developed an integrative analytical framework combining bulk transcriptomics, machine‐learning‐based biomarker prioritization, protein‐level assessment, immune profiling, single‐cell transcriptomics, spatial transcriptomics, in silico perturbation analysis, and interpretable radiogenomics to characterize two candidate biomarkers, S100B and ITGB5, in GBM. Overall, our findings support a two‐pattern interpretation of GBM heterogeneity, in which S100B reflects a relatively broad glial/tumor‐state program, whereas ITGB5 captures more focal niche‐associated remodeling involving selected malignant subpopulations, stromal–vascular compartments, and immune‐interactive regions.

S100B is a calcium‐binding protein closely associated with glial cells and has been implicated in injury responses, neuroinflammation, and stress‐related signaling. Under pathological conditions, S100B may function as a damage‐associated molecular pattern that amplifies inflammatory signaling and modulates cellular response programs through receptors such as RAGE (Moysa et al. 2021). Previous studies have also linked S100B to glioma‐associated microglial/macrophage regulation and inflammatory signaling (Zhang et al. 2011; Wang et al. 2013). In our study, S100B was prioritized because it contributed to the diagnostic models and was associated with overall survival in the analyzed cohort. At the single‐cell level, S100B was broadly expressed across glial‐lineage and malignant‐cell compartments, and pseudotime analysis suggested dynamic expression changes along inferred malignant‐cell trajectories. Spatial transcriptomics further supported a diffuse and widespread in situ distribution of S100B across the analyzed GBM tissue sections.

This broad distribution is important for interpreting the biological meaning of S100B. Its relevance in GBM may not be fully explained by a simple tumor‐versus‐normal overexpression model. In the CPTAC analysis, S100B did not show a statistically significant tumor‐normal difference in protein abundance. This finding suggests that the potential value of S100B in GBM may depend less on a uniform increase in total abundance and more on cellular distribution, malignant‐cell‐state association, and ecological context. Differences in tissue context, cohort composition, post‐transcriptional regulation, and intratumoral heterogeneity may contribute to the discordance between transcript‐level and quantitative protein‐level findings. Consistent with this interpretation, in silico S100B perturbation analysis implicated glial‐lineage‐ and tumor‐state‐related genes such as PTPRZ1 and OLIG1, together with pathways related to neurogenesis regulation, cytoskeletal remodeling, membrane organization, immune‐phagocytic processes, and metabolic adaptation. These findings support the hypothesis that S100B may be embedded in a broader glial‐lineage‐associated malignant‐cell plasticity program. Because the perturbation analysis was computational, experimental knockdown or overexpression studies are needed to determine whether S100B actively contributes to these programs or primarily reflects them.

ITGB5 showed a different biological pattern. As a member of the integrin family, ITGB5 participates in extracellular matrix adhesion, microenvironmental signal transduction, and glioma‐associated invasive behavior (Zhang et al. 2019; Zhu et al. 2020). In our study, ITGB5 had stronger cross‐platform protein‐level support than S100B, with significantly higher abundance in CPTAC proteomic data and detectable staining in HPA immunohistochemistry. Bulk transcriptomic analysis and pathway enrichment also indicated that GBM was characterized by extracellular matrix remodeling, integrin signaling, collagen‐related programs, and vascular or endothelial response pathways, consistent with the biological role of ITGB5 as an adhesion‐ and microenvironment‐associated molecule. Single‐cell and spatial transcriptomic analyses further refined this interpretation. Unlike S100B, ITGB5 was detected in only a limited fraction of cells and was concentrated in selected malignant or niche‐associated subpopulations. Pseudotime analysis suggested that ITGB5 was enriched mainly in restricted branch regions rather than broadly distributed across malignant‐cell trajectories, while spatial transcriptomics showed focal ITGB5 expression confined to localized tissue regions or scattered hotspots. Together with CellChat results linking malignant cells, TAMs, endothelial cells, and pericytes through stromal‐vascular pathways such as COLLAGEN, SPP1, FN1, VEGF, and NOTCH, these convergent computational associations are consistent with ITGB5 as a context‐dependent candidate marker of focal stromal‐immune and ECM‐remodeling niches, rather than evidence that ITGB5 causally establishes these niches.

The immune analyses provided additional context for this two‐pattern model. ssGSEA‐based immune profiling showed selective changes in GBM rather than uniform activation of all immune compartments. Tfh‐, Th2‐, and Treg‐related signatures were increased, and immune‐function analysis highlighted CCR, inflammation‐promoting activity, MHC Class I, and type II IFN responses. ITGB5 showed relatively consistent positive associations with immunoregulatory and inflammatory features, including Treg‐ and macrophage‐related signatures. S100B, in contrast, displayed a more heterogeneous immune‐correlation pattern. These findings suggest that ITGB5 may be more closely associated with focal immunoregulatory and stromal‐reactive niches, whereas S100B may reflect a broader glial‐inflammatory or tumor‐state context. This distinction is important because GBM should not be viewed simply as an immune‐desert tumor. Inflammatory activation and immune suppression may coexist within the same tumor ecosystem and may be organized in spatially and functionally distinct ways, consistent with previous descriptions of the immune‐tolerant GBM microenvironment (Kesarwani et al. 2022).

Within the microbiota‐associated conceptual framework, these findings may be biologically relevant. Previous evidence has shown that S100B can respond to systemic perturbations and influence gut vascular barrier permeability (Feng et al. 2024), while gut microbiota and their metabolites may regulate glial activity and S100B release (Feng et al. 2025). Microbiota‐derived metabolites have also been reported to modulate antitumor immune networks (Perl et al. 2025). In this context, S100B and ITGB5 may represent two different local readouts of systemic immune‐metabolic regulation: S100B may serve as a glial/tumor‐state‐associated response marker, whereas ITGB5 may mark focal stromal‐vascular and immunoregulatory niche remodeling. This interpretation requires caution. Because direct microbiome, metagenomic, metabolomic, and microbiota‐perturbation data were not included, the present study cannot establish a microbiota‐to‐GBM causal cascade. The microbiota‐related interpretation should therefore be considered hypothesis‐generating rather than mechanistically established. Even so, the observed coexistence of inflammatory activation and immune suppression is compatible with a protumorigenic and immune‐tolerant microenvironment (Kim et al. 2025).

An additional exploratory aspect of this study was the integration of molecular and imaging‐derived information. Single‐cell and spatial transcriptomic analyses provide high‐resolution biological context, whereas MRI‐derived radiomic features may capture macroscopic intratumoral heterogeneity. In this study, the S100B‐ and ITGB5‐related radiogenomic models showed different imaging‐associated patterns. The S100B‐related model retained one radiomic feature and generated a relatively simple habitat map, whereas the ITGB5‐related model retained multiple features related to gray‐level distribution, texture nonuniformity, local contrast, and energy and showed a more heterogeneous habitat pattern. This cross‐modal contrast descriptively paralleled the broader molecular distribution of S100B and the more focal ITGB5‐associated pattern, but it does not demonstrate that either gene determines the corresponding imaging phenotype. Because the internal‐validation AUCs were moderate and the performance gaps between the training and validation sets suggested possible overfitting, these results should be regarded as hypothesis‐generating imaging‐molecular associations. Independent multicenter validation, standardized imaging protocols, and ideally spatially matched imaging‐tissue studies are required before clinical interpretation or application.

## 5 Limitations

Several limitations should be acknowledged. First, although batch correction and external validation were incorporated into the bulk transcriptomic and machine‐learning workflow, residual batch effects, cohort imbalance, and platform differences cannot be completely excluded. Second, the candidate‐gene set was derived from public databases and should be interpreted as microbiota‐associated or microbiota‐relevant rather than microbiota‐driven. Third, the diagnostic models showed only moderate cross‐cohort performance, and feature selection across multiple algorithms may still carry a risk of overfitting despite the validation procedures. Fourth, the survival analyses were based mainly on Kaplan–Meier methods and univariate Cox regression in available public cohorts. Without comprehensive adjustment for established clinical and molecular covariates, including age, treatment, IDH status, and MGMT promoter methylation, S100B and ITGB5 cannot be considered independent prognostic biomarkers. Fifth, ssGSEA, inferCNV, pseudotime inference, CellChat, SingleR annotation, in silico perturbation, spatial spot annotation, and radiogenomic habitat mapping are computational inferences that generate testable hypotheses but do not replace experimental validation. Sixth, spatial transcriptomic spots may contain mixtures of cell types, and the current radiogenomic‐spatial correspondence was regional rather than stereotactically matched. Finally, the radiogenomic models were evaluated only in an internal validation set, and their generalizability remains uncertain in the absence of independent external validation.

## 6 Conclusion

S100B and ITGB5 may represent complementary biological dimensions of GBM heterogeneity. S100B appears to be associated with broadly distributed glial‐lineage and malignant‐cell‐state programs, whereas ITGB5 appears to be more closely linked to focal extracellular matrix remodeling, stromal‐vascular interactions, immunoregulatory features, and heterogeneous radiogenomic phenotypes. These findings provide a hypothesis‐generating, multilayered framework for interpreting GBM organization. Functional experiments, independent molecular cohorts, and prospective multicenter imaging studies are required to determine the reproducibility, mechanistic relevance, and potential clinical value of these associations.

## Author Contributions

**Xuhang Yang:** conceptualization, methodology, software, data curation, investigation, validation, formal analysis, supervision, visualization, project administration, resources, writing – original draft, writing – review and editing. **Yusong Zhang:** conceptualization, methodology, software, data curation, investigation, validation, formal analysis, supervision, visualization, resources. **Yutuo Zheng:** conceptualization, investigation, methodology, software, data curation. **Jian Lin:** writing – review and editing, project administration, resources, funding acquisition, writing – original draft, supervision.

## Funding

The authors have nothing to report.

## Ethics Statement

This study used publicly available, de‐identified datasets. Ethical approval and informed consent were obtained by the original studies, as reported by the corresponding repositories.

## Consent

The authors have nothing to report.

## Conflicts of Interest

The authors declare no conflicts of interest.

## Supporting information


**Figure S1:** Pan‐cancer expression and prognostic context of candidate genes.
**Figure S2:** Quality control, highly variable gene identification, and clustering parameter selection for single‐cell RNA‐seq analysis.
**Table S1:** Overview of machine‐learning algorithms, parameter settings, validation strategies, and output metrics used across diagnostic gene‐expression modeling and radiomics analyses.
**Table S2:** Imaging cohort, MRI acquisition, image–omics matching, and radiomics workflow used in the radiogenomic analysis.
**Table S3:** Software resources, package versions, and representative functions used across the analytical workflow.

## Data Availability

The transcriptomic and single‐cell RNA sequencing datasets analyzed during the current study are available in public repositories, including TCGA, CGGA, GTEx, and the Gene Expression Omnibus (GEO) database under the accession numbers GSE116520, GSE263588, GSE83300, GSE119834, GSE137900, GSE135045, and GSE237183. All custom scripts, computational pipelines, and processed analytical data underlying the results reported in this manuscript are available from the corresponding authors upon reasonable request.
