# Supplementary material for: Integrated Multi‐Omics and Radiogenomic Analysis Identifies S100B and ITGB5 as Complementary Candidate Biomarkers of Glioblastoma Heterogeneity
Source: Chem Biol Drug Des. 2026 Sep 14;108(3):e70396. doi: 10.1111/cbdd.70396 (PMC13575307; doi:10.1111/cbdd.70396)
Supplement: Supplementary file 1 [file CBDD-108-e70396-s001.docx]

**Supporting Information**

**Supplementary Figure S1. Pan-cancer expression and prognostic context of candidate genes.**

(A) Pan-cancer expression profile of ITGB5 across tumor types based on the TCGA and GTEx datasets.

(B) Pan-cancer expression profile of S100B across tumor types based on the TCGA and GTEx datasets.

(C) Heatmap of univariate Cox regression analysis showing the prognostic associations of candidate genes across multiple cancer types. Colors represent hazard ratios, and significant associations are indicated.

**Supplementary Figure S2. Quality control, highly variable gene identification, and clustering parameter selection for single-cell RNA-seq analysis.**

(A) Violin plots showing the distributions of key quality-control metrics across samples before filtering, including the number of detected genes per cell (nFeature_RNA), total UMI counts per cell (nCount_RNA), mitochondrial transcript percentage (percent.mt), and hemoglobin-related transcript percentage (percent.HB).

(B) Violin plots showing the distributions of quality-control metrics across samples after filtering.

(C) Scatter plots illustrating the correlations among major quality-control variables, including the relationship between nCount_RNA and percent.mt, and between nCount_RNA and nFeature_RNA.

(D) Identification of highly variable genes. Variable features are highlighted against all detected genes, and representative top-ranked highly variable genes are labeled.

(E) Clustree plot showing cluster stability and hierarchical relationships across different clustering resolutions, used to determine the optimal resolution for downstream analysis.

**Supplementary Table S1. Overview of machine-learning algorithms, parameter settings, validation strategies, and output metrics used across diagnostic gene-expression modeling and radiomics analyses.**

| **Algorithm** | **Primary analytical role** | **Key parameter settings** | **Internal validation strategy** | **Main output metrics** |
| --- | --- | --- | --- | --- |
| **LASSO regression** | Feature selection | alpha = 1; lambda optimized by cross-validation | 10-fold cross-validation | Selected features; AUC |
| **Ridge regression** | Feature selection | alpha = 0; lambda optimized by cross-validation | 10-fold cross-validation | Feature coefficients; AUC |
| **Elastic net** | Feature selection | alpha = 0.2, 0.4, 0.5, 0.6, 0.8; lambda optimized by cross-validation | 10-fold cross-validation | Selected features; AUC |
| **Stepwise GLM** | Feature selection | Direction = forward, backward, or both | Model-based stepwise selection within discovery cohort | Selected features; AUC |
| **glmBoost** | Feature selection | mstop = 100 | Internal resampling during training | Selected features; AUC |
| **plsRglm** | Feature selection/classification | pls-glm-logistic mode | Cross-validated fitting | Selected features; AUC |
| **Random forest** | Feature selection/classification | ntree = 1000; nodesize = 5; importance = TRUE | Model development within discovery cohort; cross-validation when applicable | AUC; feature importance |
| **GBM** | Feature selection/classification | shrinkage = 0.001; interaction.depth = 3; optimal number of trees determined by cross-validation | Cross-validation for optimal iterations | AUC |
| **XGBoost** | Feature selection/classification | max.depth = 2; eta = 1; nrounds optimized | 5-fold cross-validation | AUC; feature importance |
| **SVM** | Classification | Kernel-based classifier implemented within the training framework | 5-fold cross-validation | AUC; ROC |
| **LDA** | Classification | Default discriminant settings | Training-set fitting and comparison | AUC |
| **QDA** | Classification | Default quadratic discriminant settings | Training-set fitting and comparison | AUC |
| **Naive Bayes** | Classification | Default Bayesian classifier settings | Training-set fitting and comparison | AUC |
| **KNN** | Classification | k = 3, 5, 7, 9, 11 | 5-fold cross-validation for optimal k | AUC; ROC |
| **AdaBoost** | Classification | iter = 50; loss = logistic | Cross-validated comparison | AUC |
| **MLP** | Radiomics classification | Tuned within development cohort | Internal training/validation split | AUC; exploratory DCA |
| **ExtraTrees** | Radiomics classification | Tuned within development cohort | Internal training/validation split | AUC; exploratory DCA |

**Supplementary Table S2. Imaging cohort, MRI acquisition, image–omics matching, and radiomics workflow used in the radiogenomic analysis.**

| **Module** | **Item** | **Details** | **Purpose or note** |
| --- | --- | --- | --- |
| **Study population** | Overall cohort | A total of 92 patients with matched transcriptomic data and preoperative CE-T1WI from publicly available datasets were included in the radiogenomic analysis. | Defines the radiogenomic study population. |
| **Diagnostic framework** | Glioblastoma definition | Primary glioblastoma was classified as WHO grade IV according to the 2016 WHO Classification of Tumors of the Central Nervous System. | The 2016 WHO framework was used because the original annotations in public and retrospective cohorts were mainly based on this classification, and complete molecular information required for 2021 WHO-based reclassification was not uniformly available. |
| **Inclusion criteria** | Eligible patients | Newly diagnosed primary glioblastoma; no prior history of intracranial tumors; availability of complete clinical information; and, for multimodal analyses, availability of paired transcriptomic data and preoperative CE-T1WI. | Ensures consistency of clinical and multimodal data inclusion. |
| **Exclusion criteria** | Excluded patients | WHO grade II or III gliomas, gliomas with indeterminate grade, and cases with missing clinical information were excluded. | Reduces heterogeneity caused by uncertain diagnosis or incomplete data. |
| **Public radiomics cohort** | Model development and internal validation cohort | Ninety-two patients with paired transcriptomic data and preoperative CE-T1WI were identified for radiomics model development and internal validation. | Used for radiomics feature selection, model construction, and internal performance assessment. |
| **MRI scanners** | Scanner type and field strength | Conventional brain MRI was acquired using GE 1.5-T, GE 3.0-T, and Philips 3.0-T scanners. | Reflects the retrospective, multi-scanner nature of the imaging cohort. |
| **MRI acquisition parameters** | CE-T1WI parameters | Typical T1-weighted imaging parameters were: TR, 2000–3000 ms; TE, 7–40 ms for GE 1.5-T scanners and 20–40 ms for GE/Philips 3.0-T scanners; TI, 800–1000 ms; slice thickness, 6.0–6.5 mm. | Scanner-specific parameters were summarized as ranges because images were collected across multiple scanners and time periods. |
| **Image–omics matching** | Public radiomics cohort | Patients were included only when transcriptomic data and preoperative CE-T1WI were available from the same individual. | Ensures subject-level matching between imaging and transcriptomic data. |
| **ROI segmentation** | Segmentation software and readers | Tumor ROIs were manually delineated on CE-T1WI using ITK-SNAP software, version 4.10.2, by two experienced radiologists blinded to clinical information and outcomes. | Reduces bias from clinical or outcome information during segmentation. |
| **Segmentation disagreement** | Consensus review | Discrepancies in segmentation were reviewed and resolved by consensus. | Improves consistency of final tumor masks. |
| **Image preprocessing** | Resampling | All CE-T1WI images were resampled to isotropic voxels of 1 × 1 × 1 mm³. | Reduces heterogeneity caused by differences in spatial resolution. |
| **Image preprocessing** | Bias field correction | Intensity nonuniformity caused by radiofrequency field inhomogeneity was corrected using the N4 bias field correction algorithm. | Reduces low-frequency intensity inhomogeneity. |
| **Image preprocessing** | Intensity normalization | Image intensity normalization was performed before radiomics feature extraction. | Improves comparability across images. |
| **Image preprocessing** | Gray-level discretization | Gray-level discretization was performed using a fixed bin width of 5. | Standardizes gray-level quantization before texture feature extraction. |
| **Feature reproducibility** | Interobserver reproducibility | Radiomics feature reproducibility was evaluated using ICC. Features with ICC values greater than 0.75 were retained. | Reduces the influence of interobserver variability in tumor delineation. |
| **Feature standardization** | Z-score normalization | All extracted radiomics features were standardized using z-score normalization before model development. | Ensures comparable feature scales during model construction. |
| **Radiomics feature extraction** | Software workflow | Radiomics feature extraction was performed using a standardized workflow based on PyRadiomics/OnekeyAI; PyRadiomics and OnekeyAI versions were not recorded. | Provides the radiomics extraction framework while transparently reporting unavailable version information. |
| **Radiomics feature extraction** | Image inputs | Features were extracted from original CE-T1WI images and filtered images, including Laplacian of Gaussian and wavelet-filtered images. | Captures both original and filtered imaging phenotypes. |
| **Radiomics feature categories** | Extracted feature types | Extracted features included shape, first-order statistics, gray-level co-occurrence matrix, gray-level run-length matrix, gray-level size-zone matrix, gray-level dependence matrix, and neighboring gray-tone difference matrix features. | Covers intensity, morphology, and texture-based tumor heterogeneity features. |
| **Initial feature number** | Radiomics feature set | A total of 1,197 radiomics features were initially extracted from each tumor ROI before feature selection. | Defines the initial candidate feature pool. |
| **Dataset splitting** | Internal training and validation | The radiogenomic cohort of 92 patients was randomly divided into a training set and an internal validation set at a ratio of 7:3. | Used for model development and internal validation. |
| **Reproducibility** | Random seed | The random seed was set to 0. | Ensures reproducibility of dataset splitting and model development. |
| **Model development** | Feature selection and training | Feature selection, model construction, and hyperparameter tuning were performed exclusively within the training set. | Reduces the risk of information leakage. |
| **Feature selection** | LASSO regression | Radiomics feature selection and Rad-score construction were performed using least absolute shrinkage and selection operator regression. | Generates a radiomics signature based on selected features. |
| **Classifier construction** | Machine-learning classifiers | Multilayer perceptron, random forest, and ExtraTrees classifiers were constructed based on selected features. | Evaluates multiple machine-learning strategies for radiomics classification. |
| **Model evaluation** | Performance metrics | ROC curves and AUC values were used to evaluate model performance in the internal validation set. Decision curve analysis was used as an exploratory assessment of model-derived net benefit. | Assesses model discrimination and exploratory decision-curve performance. |
| **Habitat mapping** | Voxel-wise analysis | Voxel-level radiomics features were calculated from CE-T1WI using tumor segmentation masks and linearly combined using coefficients from the predefined radiogenomic model to generate voxel-wise habitat score maps. | Visualizes intratumoral imaging heterogeneity. |
| **Interpretation** | Radiogenomic inference | Radiogenomic and habitat mapping analyses were interpreted as exploratory associations between imaging phenotypes and molecular characteristics. | Avoids overinterpretation as direct causal evidence that gene expression determines MRI phenotypes. |

**Supplementary Table S3. Software resources, package versions, and representative functions used across the analytical workflow.**

| **Analysis module** | **Software/package** | **Version** | **Representative functions or implementation details** |
| --- | --- | --- | --- |
| **Bulk preprocessing and batch correction** | R; limma | R 4.5.2; limma 3.64.3 | removeBatchEffect; linear-model-based analysis and batch-effect correction |
| **Differential expression analysis** | limma | 3.64.3 | lmFit, eBayes, topTable |
| **Functional enrichment analysis** | clusterProfiler; org.Hs.eg.db; ReactomePA; aPEAR | clusterProfiler v4.16.0; version information for org.Hs.eg.db, ReactomePA, and aPEAR could not be recovered from the archived analysis environment | enrichGO, enrichKEGG, enrichDO, enrichPathway, GSEA; enrichment clustering |
| **Candidate target retrieval and interaction analysis** | GeneCards; gutMGene; GeneMANIA | Web resources | Candidate gene retrieval, intersection analysis, interaction-network construction |
| **Immune infiltration analysis** | R; GSVA; GSEABase; limma | GSVA v1.50.0; GSEABase v1.64.0; limma v3.58.1 | ssGSEA scoring of immune-cell and immune-function signatures, immune-feature matrix generation, Wilcoxon rank-sum test, Spearman correlation analysis, and Benjamini–Hochberg correction |
| **Diagnostic machine learning** | Main ML packages listed in Methods Section 2.3 | R 4.5.2; exact ML package versions reported in main Methods Section 2.3 | Model training, feature selection, ROC/AUC analysis, visualization |
| **SHAP-based model interpretability** | R; caret; randomForest; kernlab; xgboost; klaR; gbm; pROC; kernelshap; shapviz; DALEX | caret v7.0-1; pROC v1.19.0.1; version information for the remaining packages could not be recovered from the archived analysis environment | Stratified 70:30 training/internal-validation split using createDataPartition; random seed = 12345; 5-fold resampling; 5-fold cross-validation; kernel SHAP calculation; SHAP bar, beeswarm, dependence, heatmap, waterfall, and force plots; permutation feature importance |
| **Survival analysis** | survival; survminer | survival v3.8-3; survminer v0.5.2 | survfit, coxph, ggsurvplot |
| **Pan-cancer validation** | GEPIA2 | Web resource | Cross-cancer expression and survival assessment |
| **scRNA-seq preprocessing and clustering** | R; Seurat; harmony | Seurat v5.4.0; harmony v2.0.3 | Quality control, normalization, variable-feature selection, scaling, PCA, Harmony-based batch correction, clustering, UMAP/t-SNE visualization |
| **Malignant-cell identification** | R; inferCNV; Seurat | inferCNV v1.16.0; Seurat v5.4.0 / v4.4.0 according to the corresponding analysis environment | CNV inference and putative malignant-cell identification |
| **Cell-cell communication analysis** | R; CellChat; Seurat; future; patchwork | CellChat v1.6.1; Seurat v4.4.0; future v1.33.1; patchwork v1.1.3 | computeCommunProb, aggregateNet, netAnalysis_signalingRole |
| **In silico knockout analysis** | scTenifoldKnk | scTenifoldKnk v1.0.3 | scGRN construction, virtual S100B knockout, manifold alignment, and identification of predicted network-level changes |
| **Protein-level validation** | Human Protein Atlas; CPTAC resource | Web resources | Review of representative HPA immunohistochemical images and comparison of normalized CPTAC proteomic expression data |
| **Spatial transcriptomics** | R; Seurat; SingleR; celldex; hdf5r; ggplot2; patchwork | Seurat v4.4.0; SingleR v2.4.1; celldex v1.12.0; hdf5r v1.3.8; ggplot2 v3.4.4; patchwork v1.1.3; glmGamPoi not available in the current environment | Read10X_h5, Read10X_Image, SCTransform, PCA, clustering, UMAP, FindAllMarkers, FindSpatiallyVariableFeatures with Moran’s I, spatial feature plotting, and SingleR-based auxiliary spot annotation |
| **Radiomics segmentation and modeling** | ITK-SNAP; Python radiomics/machine-learning workflow | ITK-SNAP v4.10.2; version information for Python and associated packages could not be recovered from the archived analysis environment | Manual ROI segmentation on preoperative CE-T1WI MRI, feature extraction, LASSO screening, classifier construction |
| **Voxel-wise habitat mapping** | SimpleITK; PyRadiomics | Version information could not be recovered from the archived analysis environment | CE-T1WI image/mask loading, resampling, voxel-wise feature extraction, habitat-score map generation |
